# Supplementary material for: Programmed folding into spiro-multicyclic polymer topologies from linear and star-shaped chains
Source: Commun Chem. 2020 Aug 4;3:97. doi: 10.1038/s42004-020-00355-4 (PMC9814586; doi:10.1038/s42004-020-00355-4)
Supplement: Supplementary file 2 — Supplementary Information [file 42004_2020_355_MOESM2_ESM.pdf]

Supplementary Information

Programmed folding into *spiro*-multicyclic polymer topologies from linear and star-shaped chains

Mato, Y. et al.

S1. Supplementary Methods

S1-1. Materials

Grubbs' catalyst 3rd generation (G3),[1] 1,3-bis((1-methyl-1,1-bis(hydroxymethyl))-2-ethoxy)-2-((*p*-methoxybenzoxy)methyl)propane (**I3**),[2] and 4-(hydroxymethyl)styrene[3] were prepared according to previously reported method. Amberlyst® A21 (Organo Co., Ltd), *N*,*N*-dimethyl-4-aminopyridine (DMAP; Tokyo Chemical Industry Co., Ltd. (TCI), >99.0%), 1-ethyl-3-(3-(dimethylamino)-propyl)carbodiimide hydrochloride (EDC; TCI, >98.0%), ethyl vinyl ether (TCI, >98.0%), 2,3-dichloro-5,6-dicyano-1,4-benzoquinone (DDQ; TCI, >97.0%), *p*-methoxybenzyl chloride (PMBCl; TCI, >98.0%), (±)-*exo*-5-norbornenecarboxylic acid (*exo*-NB-COOH; Aldrich, 97%), *cis*-2-butene-1,4-diol (TCI, >94.0%), *t*-Bu-P4 (in hexane as ~0.8 mol L^{-1} solution; Sigma-Aldrich Chemicals), sodium iodide (NaI; Wako Pure Chemical Industry Co. Ltd., >99.5%), and 2, 5-dihydroxybenzoic acid (DHB; Sigma-Aldrich, >98.0%) were used as received. *ε*-Caprolactone (*ε*-CL; TCI, >99%), 2-ethylhexyl glycidyl ether (TCI, >98.0%), 1,8-diazabicyclo[5.4.0]-7-undecene (DBU; TCI, >98.0%) were purified by distillation over CaH_2 under reduced pressure and stored in the glovebox. L-Lactide (TCI, >98.0%) was purified twice by recrystallization using dry toluene and stored in the glovebox. Diphenyl phosphate (DPP; TCI, >99.0%) and synthesized initiators were purified by azeotropic distillation with dry toluene and stored in the glovebox.

S1-2. Instruments

The polymerization experiments were carried out in an MBRAUN stainless steel glovebox equipped with a gas purification system (molecular sieves and copper catalyst) in a dry argon atmosphere (H_2O, O_2<0.1 ppm). The moisture and oxygen contents in the glovebox were monitored by an MB-MO-SE 1 moisture sensor and an MB-OX-SE 1 oxygen sensor, respectively. Dry toluene (>99.5%; water content, <0.001%, Kanto Chemical Co., Inc.) used for the polymerization was purified by passing through an MBRAUN solvent purification system (MB SPS COMPACT) consisting of a column of activated alumina and a column with activated copper catalyst. The ^{1}H (400 MHz) and ^{13}C NMR (100 MHz) spectra were recorded using a JEOL JNM-ECS400 instrument at room temperature in $CDCl_3$ or methanol-d_4. The size exclusion chromatography (SEC) was performed at 40 °C in THF (flow rate, 1.0 mL min^{-1}) using a Shodex GPC-101 gel permeation chromatography system (Shodex DU-2130 dual pump, Shodex RI-71-S reflective index detector, and Shodex ERC-3125SN degasser) equipped with a Shodex KF-G guard column (4.6 mm × 10 mm; particle size, 8 μm) and two Shodex KF-804L columns (linear, 8 mm × 300 mm) or a Jasco high-performance liquid chromatography system (PU-980 Intelligent HPLC Pump, CO-2065 Plus Intelligent Column Oven, RI-2031 Plus Intelligent RI Detector, and DG-2080-53 Degasser) equipped with a Shodex KF-G guard column (4.6 mm × 10 mm; particle size, 8 μm) and two Shodex KF-804L columns (linear; particle size 7 μm; 8.0 mm × 300 mm; exclusion limit, 4 × 10^{4}). The number-average molecular weight ($M_{n,SEC}$) and the dispersity ($Đ$) of the polymers were calculated on the basis of polystyrene calibrations. The absolute

weight-averaged molecular weights ($M_{w,MALS}$) of the samples were determined by SEC with multiangle light scattering detection (SEC-MALS-Visco) in THF (flow rate, 1.0 mL min^{-1}) at 40 °C using an Agilent 1100 series instrument equipped with a DG 1100 degasser, a Shodex KF-G guard column (4.6 mm × 10 mm; particle size, 8 μm), a Shodex KF-800D solvent-peak separation column (linear, 8.0 mm × 100 mm; particle size, 10μm), two Shodex KF-805L columns (linear, 8.0 mm × 300 mm; exclusion limit, 4.0 × 10^{6}; particle size, 10 μm), a DAWN 8+ multiangle laser light scattering detector (Wyatt Technology), an Optilab rEX refractive index detector (Wyatt Technology), and a Viscostar viscosity detector (Wyatt Technology). The preparative SEC for Grubbs' catalyst removal was performed at r.t. in $CHCl_3$ (flow rate, 3.5 mL min^{-1}) using LC-9201 liquid chromatography system (Japan Analytical Industry Co. Ltd.) equipped with a BG-12 degasser, a PI-50 pump, a RI-50S RI detector, a JAIGEL-H-P guard column (8 mm × 40 mm; Japan Analytical Industry Co. Ltd.), and a Shodex K-2004 column (linear, 20.0 mm × 300 mm; exclusion limit, 1.4 × 10^{4}; particle size, 7 μm). The matrix-assisted laser desorption ionization time-of-flight mass spectrometry (MALDI-TOF MS) of the obtained polymers was performed using an Applied Biosystems Voyager-DE STR-H equipped with a 337 nm nitrogen laser (3 ns pulse width). Two hundred shots were accumulated for the spectra at a 20 kV acceleration voltage in the reflector mode and calibrated using PSt as the internal standard. Samples for the MALDI-TOF MS were prepared as follows: (i) the THF solution of polymer sample (4.0 mg mL^{-1}) and the THF solution of matrix (DHB; 60 mg mL^{-1}) were mixed at a volume ratio of 1:1. (ii) Then, the sample plate was spotted by the THF solution of cationic agent (NaI; 1.0 mg mL^{-1},

1.0 μL), followed by the mixed solution (1.0 μL). The thermal properties of the polymer samples were measured from −50 to 100 °C during the second heating by a Bruker AXS DSC 3100 differential scanning calorimeter under a nitrogen atmosphere with the heating rate of 10 °C min^{-1} and cooling rate 20 °C min^{-1}. The melting temperature (T_m) was determined as peak maxima of transition during 2nd heating run. Thermogravimetric analysis (TGA) was performed from 20 to 500 °C with a heating rate of 10 C min^{-1} by a Bruker AXS DSC 3100 under a nitrogen atmosphere. Synchrotron small-angle X-ray scattering (SAXS) and wide-angle X-ray diffraction (WAXD) measurements of the obtained polymers were performed with an X-ray beam of 1.5Å at the BL-6A in the Photon Factory (Tsukuba, Japan). The 2D SAXS and WAXD profiles were obtained with a Pilatus 1M and 100K detectors, respectively, which were circularly averaged to produce the 1D plots of log I (intensity) and q (scattering vector). The q value was calibrated using a silver behenate. The powder sample of the polymer was put into a Hilgenberg lindemann glass capillary (1.5 mm × 80 mm), which was annealed at 100 °C for 1 h in a pre-heated oven to erase a thermal history and then cooled to room temperature. The crystallinity (X_{WAXD}) was determined by the peak deconvolution of WAXD profile.

S1-3. Synthetic details

Synthesis of 2-ethyl-2-(((4-methoxybenzyl)oxy)methyl)propane-1,3-diol (I1)

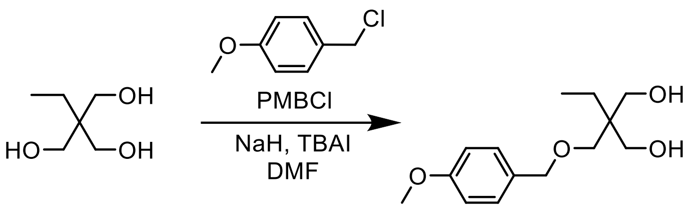

I1

A typical procedure for the protection with *p*-methoxybenzyl chloride is as follows (method A): Under an argon atmosphere, trimethylolpropane (34.8 g, 259 mmol) was added to a solution of NaH (3.84 g, 96.0 mmol; 60% in mineral oil) and tetrabutylammonium iodide (TBAI; 2.59 g, 7.01 mmol) in DMF (400 mL), then the solution was stirred at 70 °C for 30 min. A solution of *p*-methoxybenzyl chloride (PMBCl; 10.0 g, 63.9 mmol) in DMF (100 mL) was added dropwise to the reacting mixture, and the resulting mixture was stirred at 70 °C for 24 h under an argon atmosphere. After removing the solvent by evaporation, the obtained residue was dissolved in AcOEt and washed with brine. The organic layer was dried over anhydrous Na_2SO_4, filtrated, and then concentrated. The residue was purified by silica gel column chromatography (AcOEt/*n*-hexane = 8/2, R_f = 0.30) to give **I1** as a white solid. (11.1 g). Yield: 68.3%

^{1}H NMR (400 MHz, $CDCl_3$): δ(ppm) 7.23 (d, 2H, J = 8.70, aromatic), 6.87 (d, 2H, J = 8.70, aromatic), 4.42 (s, 2H, -PhCH_2O-), 3.80 (s, 3H, -CCH$_2$CH_3), 3.68 (d, 2H, J = 11.0, -OCH_2C-), 3.57 (d, 2H, J = 11.0, -CCH_2OH), 3.43 (s, 2H, -CCH_2CH$_3$), 2.90 (s, 2H, -OH), 0.80 (t, 3H, J = 7.50, -OCH$_3$). HRMS (ESI, in methanol solution): *m/z* calcd for $C_{14}H_{22}O_4Na$: 277.1410 $[M+Na]^+$; found: 277.1410.

Synthesis of PMBO-(PCL-OH)$_2$

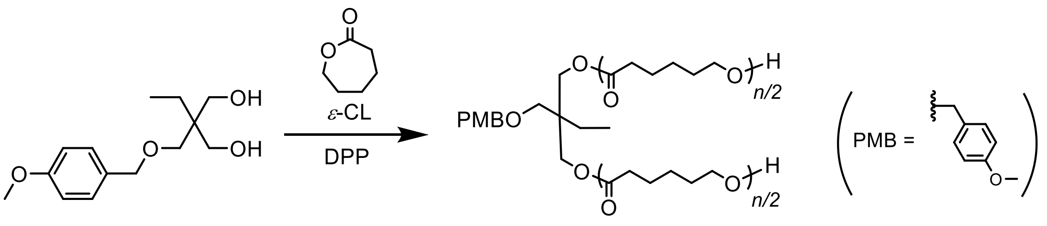

I1 PMBO-(PCL-OH)$_2$

A typical procedure for the polymerization is as follows (method B): Under an argon atmosphere, ε-CL (3.00 g, 26.3 mmol) was added to a solution of **I1** (134 mg, 525 μmol) in toluene (20.2 mL) at r.t. in a reaction vessel. Diphenyl phosphine (DPP; 132 mg, 525 μmol) was then added to the solution to initiate the polymerization. After 3 h, the polymerization was quenched by the addition of Amberlyst® A21. The polymer crude was purified by the reprecipitation from CH_2Cl_2 into cold methanol/*n*-hexane (v/v = 10/1) to give PMBO-(PCL-OH)$_2$ as a white solid. (2.49 g) Yield: 89.3%

$M_{n,NMR}$ = 5,670 g mol^{-1}, $M_{n,SEC}$ = 9,640 g mol^{-1}, $Đ$ = 1.06

^{1}H NMR (400 MHz, $CDCl_3$): δ (ppm) 7.21 (d, J = 8.70, aromatic), 6.86 (d, J = 8.70, aromatic), 4.39 (s, -PhCH_2O-), 4.15-3.93 (m, -OCO(CH_2)$_4$CH_2-), 3.80 (s, -CCH$_2$CH_3), 3.69-3.61 (m, -CH_2OH), 3.30 (d, J = 8.20, -OCH_2C-), 2.43-2.22 (m, -OCOCH_2(CH_2)$_4$-), 1.86-1.52 (m, -OCOCH$_2$CH_2(CH_2)$_3$-, -OCO(CH_2)$_3$CH_2CH$_2$-), 1.50-1.24 (m, -OCO(CH_2)$_2$CH_2(CH_2)$_2$-, -CH_2CH$_3$), 0.84 (t, J = 7.50 Hz, -CH$_2$CH_3).

Synthesis of HO-(PCL-OH)$_2$

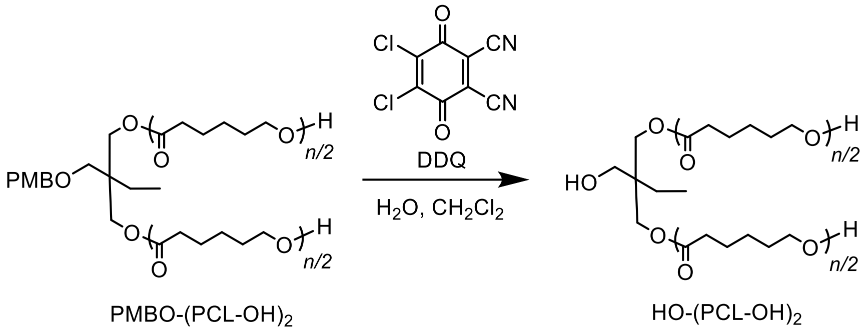

A typical procedure for the deprotection reaction is as follows (Method C): 1,2-Dichloro-4,5-dicyano-*p*-benzoquinone (DDQ; 161 mg, 708 mmol) was added to the solution of PMBO-(PCL-OH)$_2$ ($M_{n,NMR}$ = 5,670 g mol^{-1}, 2.0 g, 351 μmol) in CH_2Cl_2/water (30.0 mL, v/v = 2/1), and the reaction mixture was stirred at r.t. for 24 h. The polymer crude was purified by reprecipitation twice from CH_2Cl_2 into cold methanol to give HO-(PCL-OH)$_2$ as a white solid (1.04 mg). Yield: 52.0%

$M_{n,NMR}$ = 5,650 g mol^{-1}, $M_{n,SEC}$ = 9,690 g mol^{-1}, $Đ$ = 1.06

^{1}H NMR (400 MHz, $CDCl_3$): δ (ppm) 4.31-3.83 (m, -OCO(CH_2)$_4$CH_2-), 3.66 (t, J = 6.40, -(CH_2)$_4$CH_2OH), 3.42 (d, J = 6.40, -CCH_2OH), 2.54-2.11 (m, -OCOCH_2(CH_2)$_4$-), 1.90-1.53 (m, -OCOCH$_2$CH_2(CH_2)$_3$-, -OCO(CH_2)$_3$CH_2CH$_2$-), 1.52-1.32 (m, -OCO(CH_2)$_2$CH_2(CH_2)$_2$-, -CCH_2CH$_3$), 0.90 (t, J = 7.50, -CCH$_2$CH_3).

2.6 Synthesis of P2-a

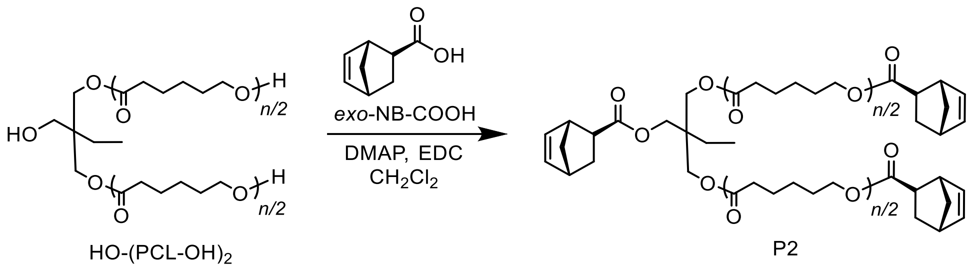

A typical procedure for the condensation reaction is as follows (method D): In a Schlenk flask, HO-PCL-OH ($M_{n,NMR}$ = 5,700 g mol^{-1}, 1.0 g, 175 μmol), *exo*-NB-COOH (147 mg, 1.06 mmol), DMAP (195 mg, 159 mmol), and EDC (305 mg, 1.59 mmol) were dissolved in CH_2Cl_2 (10 mL) and the mixture was stirred at r.t. for 24 h. The polymer crude was purified by reprecipitation twice from CH_2Cl_2 into cold methanol to give **P2-a** as a white solid. (533 mg) Yield: 55.3%

$M_{n,NMR}$ = 6,200 g mol^{-1}, $M_{n,SEC}$ = 9,970 g mol^{-1}, $Đ$ = 1.06

^{1}H NMR (400 MHz, $CDCl_3$): δ (ppm) 6.19-6.02 (m, -C*H*=C*H*- in norbornene ring), 4.32-3.77 (m, -OCO(CH_2)$_4$C*H*$_2$-), 3.08-2.95 (m, -C*H*-CH-CH_2O- in norbornene ring), 2.90 (s, -C*H*-CH-CH_2O- in norbornene ring), 2.53-2.06 (m, -OCOC*H*$_2$(CH_2)$_4$-), 2.00-1.83 (m, -OCO(CH_2)$_2$C*H*$_2$(CH_2)$_2$-, bridge head -CH_2- in norbornene ring, *endo*-C*H*- of -CH-C*H*$_2$-CH-CH_2O-), 1.80-1.51 (m, -OCOCH$_2$C*H*$_2$(CH_2)$_3$-, -OCO(CH_2)$_3$C*H*$_2$CH$_2$-), 1.45-1.21 (-OCO(CH_2)$_2$C*H*$_2$(CH_2)$_2$-, -CC*H*$_2$CH$_3$), 1.01-0.79 (t, J = 7.50, -CCH$_2$C*H*$_3$)

Synthesis of MC2-a (8-shaped PCL)

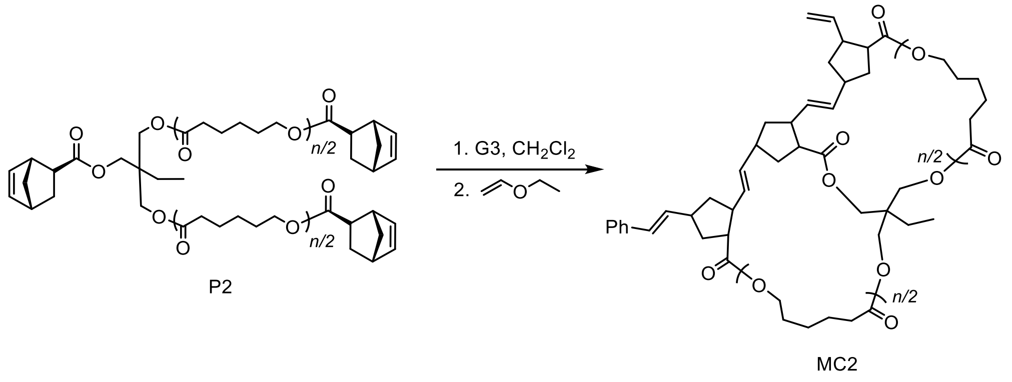

A typical procedure for the intramolecular ring-opening metathesis oligomerization is as follows (method E): G3 (25.7 mg, 29.0 µmol) was added to a three-necked flask and dissolved in deggased-CH_2Cl_2 (240 mL). Then, a solution of **P2-a** ($M_{n,NMR}$ = 6,200 g mol^{-1}, 30.0 mg, 4.84 µmol, 170 µM in CH_2Cl_2) was added dropwise to the G3 solution through the additional funnel over 20 min. After 5 min, the reaction was quenched by the addition of ethyl vinyl ether (900 µL). The metal residue in the crude product was removed by preparative SEC (solvent, $CHCl_3$) to give **MC2-a** as a pale brown solid (27.8 mg). Yield: 91.0%

$M_{n,SEC}$ = 7,300 g mol^{-1}, $Đ$ = 1.08

^{1}H NMR (400 MHz, $CDCl_3$): δ(ppm) 6.62-1.88 (br, alkenyl of poly(norbornene) backbone), 4.37-3.81 (m, -OCO(CH_2)$_4$CH_2-), 2.38-2.22 (m, -OCOCH_2(CH_2)$_4$-), 1.73-1.57 (m, -OCOCH$_2$CH_2(CH_2)$_3$-, -OCO(CH_2)$_3$CH_2CH$_2$-), 1.44-1.22 (m, -OCO(CH_2)$_2$CH_2(CH_2)$_2$-, -CCH_2CH$_3$), 0.90-0.82 (m, -CCH$_2$CH_3).

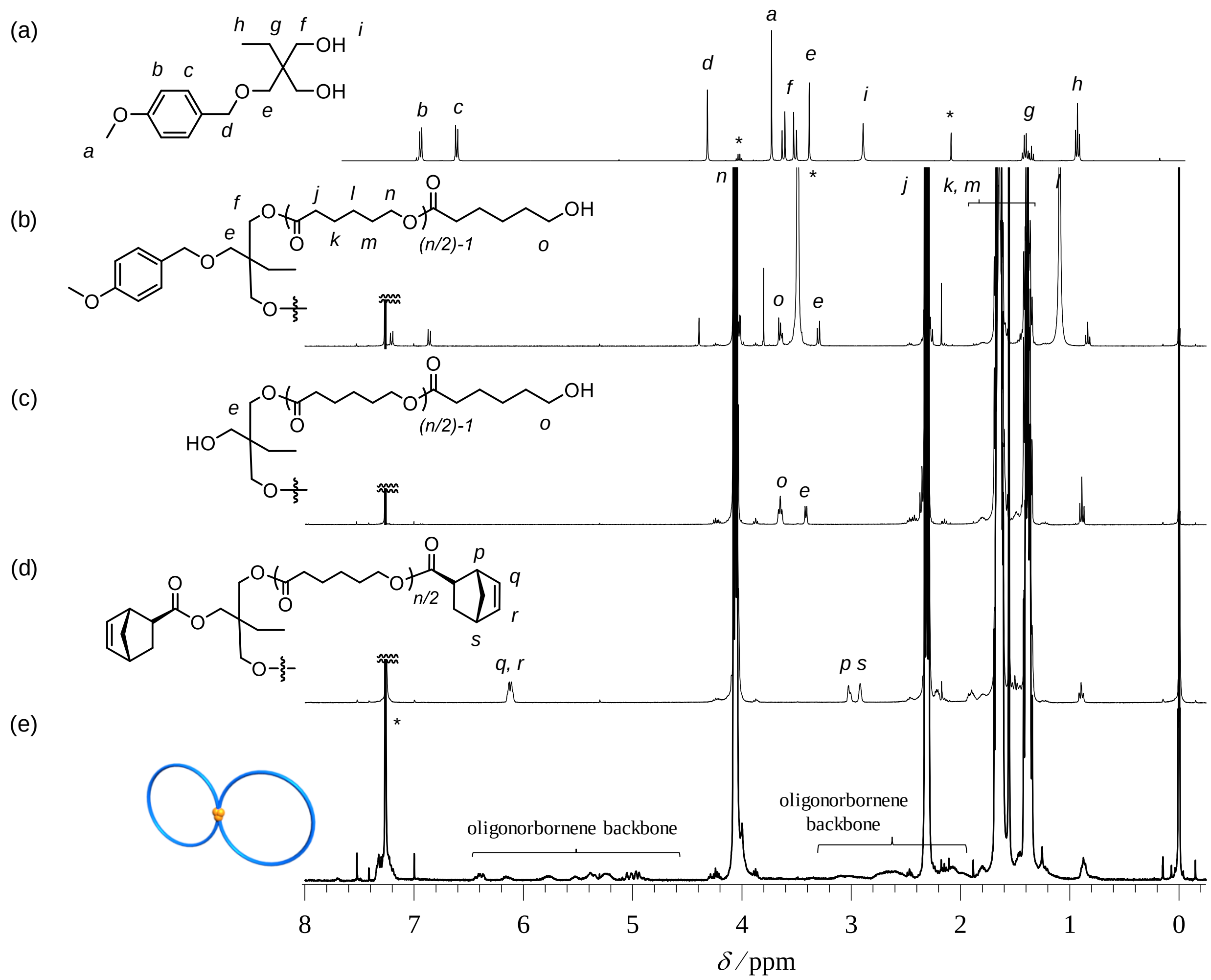

Supplementary Figure 1. NMR spectra of (a) **I1**, (b) PMBO-(PCL-OH)$_2$, (c) HO-(PCL-OH)$_2$, (d) **P2-a**, and (e) **MC2-a** in $CDCl_3$ (400 MHz).

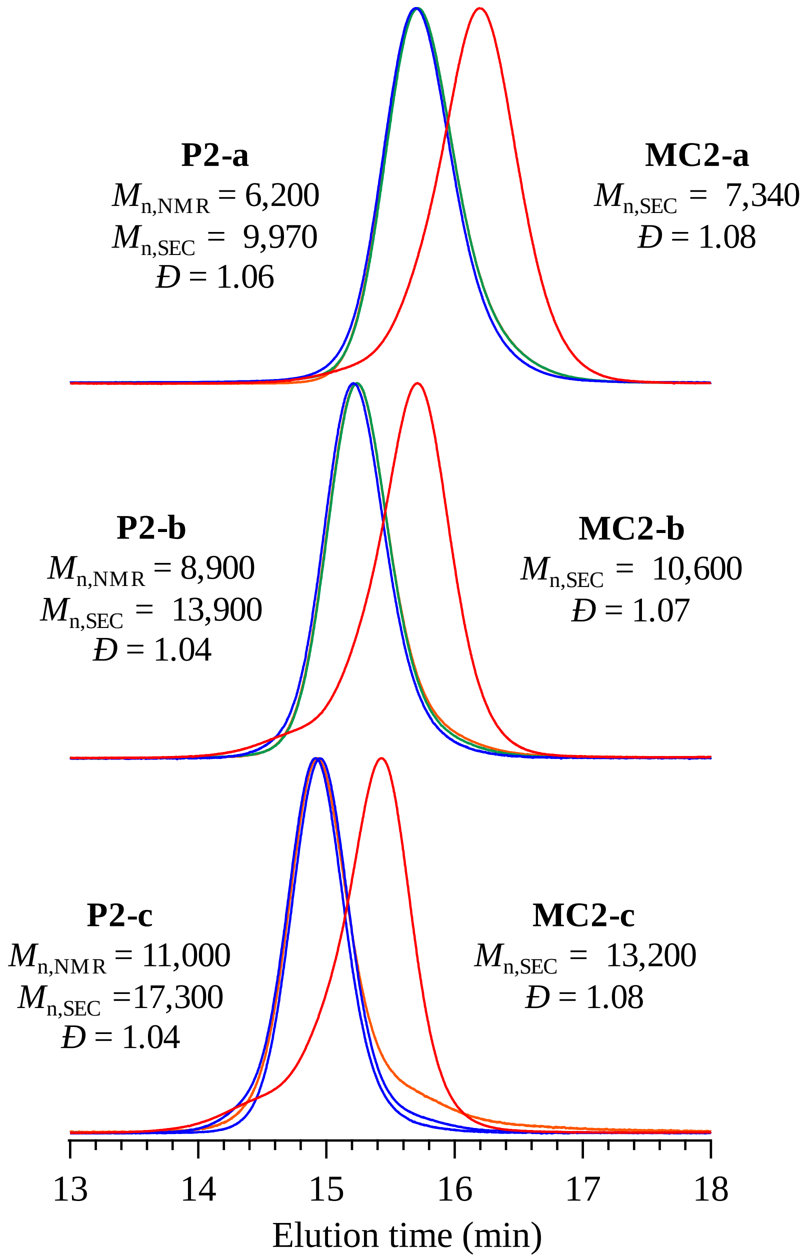

Supplementary Figure 2. SEC traces of PMBO-(PCL-OH)$_2$s (orange), HO-(PCL-OH)$_2$s (green), **P2**s (blue) and **MC2**s (red) with different molecular weight.

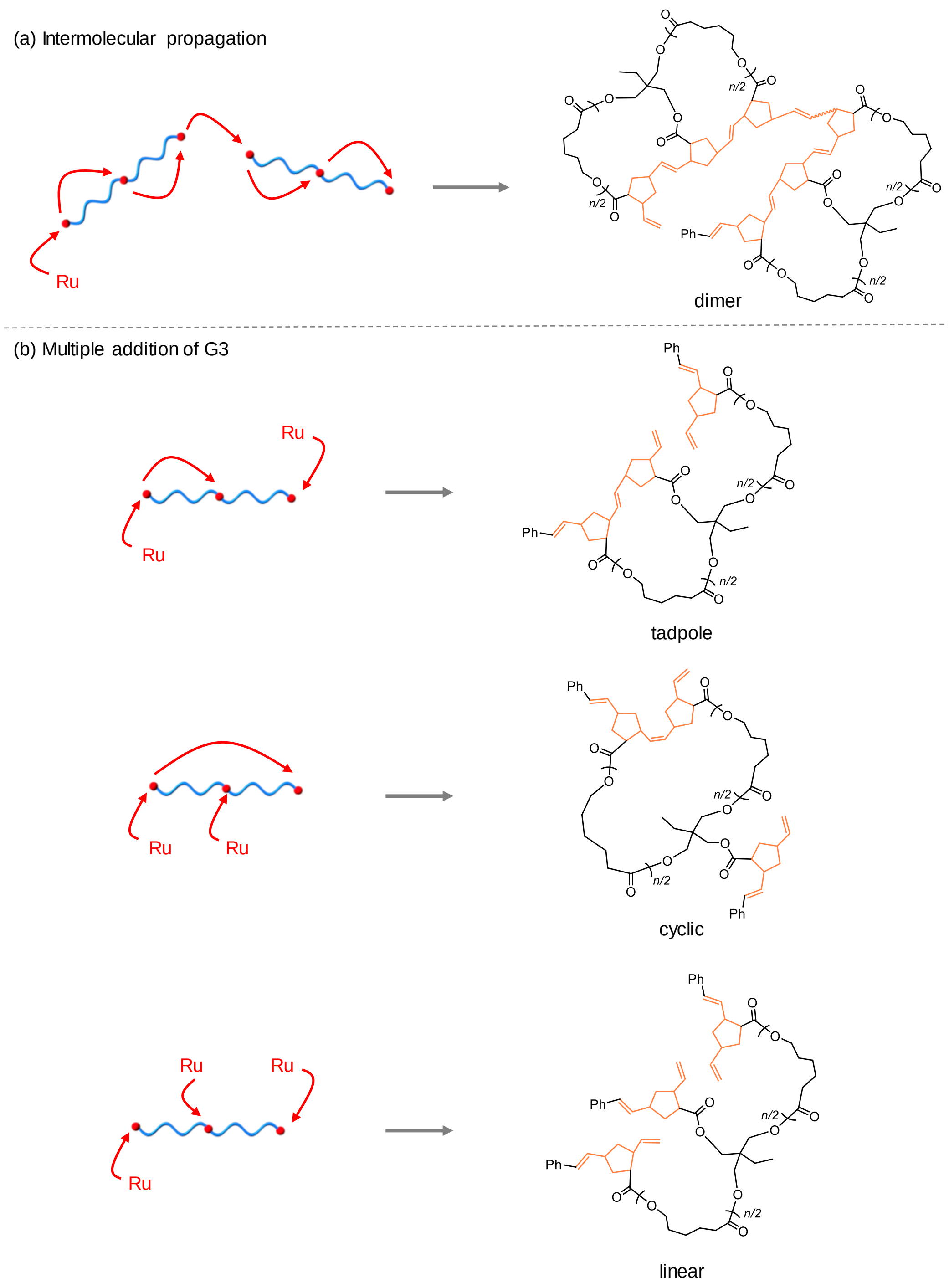

Supplementary Figure 3. Schematic illustration of possible side reactions during the synthesis of **MC2**: (a) oligomer formation by intermolecular propagation and (b) by-product formations through multiple addition of G3.

Supplementary Table 1. Molecular characterization of **MC2**s and their precursors

Sample	$M_{n,NMR}$ [a]	$M_{n,SEC}$ [b]	$M_{w,MALS}$ [c]	$Đ$ [b]	yield (%)	D_h [c] (nm)	$[\eta]$ [c] (mL g^{-1})	T_m [d] (°C)	X_{WAXD} [e] (%)
PMBO-$(PCL\text{-}OH)_2$-a	5,670	9,640	-	1.06	89.3	-	-	-	-
HO-$(PCL\text{-}OH)_2$-a	5,650	9,690	-	1.06	52.0	-	-	-	-
P2-a	6,200	9,970	6,210	1.06	55.3	5.0	18.3	47.9	45.1
MC2-a	-	7,340	5,190	1.08	91.0	4.2	9.5	53.8	41.8
PMBO-$(PCL\text{-}OH)_2$-b	8,000	13,400	-	1.04	88.1	-	-	-	-
HO-$(PCL\text{-}OH)_2$-b	7,900	13,500	-	1.04	64.4	-	-	-	-
P2-b	8,900	13,900	6,940	1.04	76.2	5.8	22.1	51.4	45.2
MC2-b	-	10,600	8,770	1.07	89.7	5.0	12.3	56.1	44.5
PMBO-$(PCL\text{-}OH)_2$-c	10,600	15,700	-	1.07	63.3	-	-	-	-
HO-$(PCL\text{-}OH)_2$-c	10,600	16,600	-	1.04	81.8	-	-	-	-
P2-c	11,000	17,300	9,810	1.04	70.0	6.6	26.5	52.8	40.3
MC2-c	-	13,200	12,700	1.08	86.7	5.6	14.9	57.7	50.6

[a] Determined by ^{1}H NMR. [b] Determined by SEC in THF using PSt standards. [c] Determined by SEC-MALS-Visco in THF. [d] Determined from a melting peak of the DSC curve. [e] Determined by WAXD at r.t..

Synthesis of 2-(hydroxymethyl)-2-(((4-methoxybenzyl)oxy)methyl)propane-1,3-diol (I2)

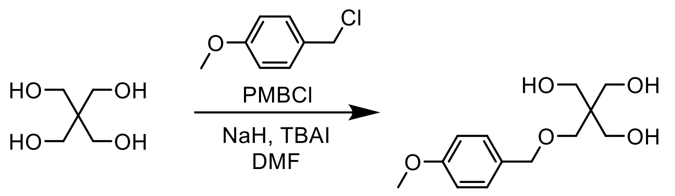

I2

Method A was used for the protection of pentaerythritol (34.8 g, 255 mmol) with PMBCl (10.0 g, 63.9 mmol), NaH (3.84 g, 96 mmol; 60% in mineral oil), and TBAI (2.59 g, 7.01 mmol) in DMF (500 mL) to give **I2** as a white solid (11.1 g). Yield: 67.9%

^{1}H NMR (400 MHz, $CDCl_3$): δ (ppm) 7.25-7.15 (m, aromatic), 6.90-6.83 (m, aromatic), 4.41 (d, J = 12.8, -PhCH_2O-), 4.29-3.85 (m, -OCO(CH_2)$_4$CH_2-), 3.80 (d, J = 1.80, -OCH_3), 3.65 (t, J = 6.60, -CH_2OH), 3.42 (d, J = 18.7, -PhCH$_2$OCH_2-), 2.40-2.22 (m, -OCOCH_2(CH_2)$_4$-), 1.79-1.52 (m, -OCOCH$_2$CH_2(CH_2)$_3$-, -OCO(CH_2)$_3$CH_2CH$_2$-), 1.47-1.28 (-OCO(CH_2)$_2$CH_2(CH_2)$_2$-).

Synthesis of PMBO-(PCL-OH)$_3$

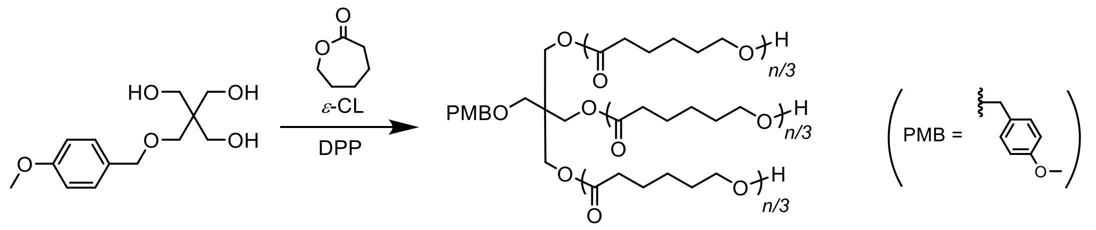

I2 PMBO-(PCL-OH)$_3$

Method B was used for the polymerization of ε-CL (3.0 g, 26.3 mmol) with PMBO-OH$_3$ (135 mg, 525 μmol), and DPP (132 mg, 525 μmol) in toluene (20.2 mL) at r.t. for 2.7 h to give PMBO-(PCL-OH)$_3$ as white solid (2.65 g). Yield: 88.3%

$M_{n,NMR}$ = 4,820 g mol^{-1}, $M_{n,SEC}$ = 8,190 g mol^{-1}, $Đ$ = 1.08

^{1}H NMR (400 MHz, CDCl$_3$): δ (ppm) 7.25-7.15 (m, aromatic), 6.90-6.83 (m, aromatic), 4.41 (d, J = 12.8, -PhCH_2O-), 4.29-3.85 (m, -OCO(CH$_2$)$_4$CH_2-), 3.80 (d, J = 1.80, -OCH_3), 3.65 (t, J = 6.60, -CH_2OH), 3.42 (d, J = 18.7, -PhCH$_2$OCH_2-), 2.40-2.22 (m, -OCOCH_2(CH$_2$)$_4$-), 1.79-1.52 (m, -OCOCH$_2$CH_2(CH$_2$)$_3$-, -OCO(CH$_2$)$_3$CH_2CH$_2$-), 1.47-1.28 (m, -OCO(CH$_2$)$_2$CH_2(CH$_2$)$_2$-).

Synthesis of HO-(PCL-OH)$_3$

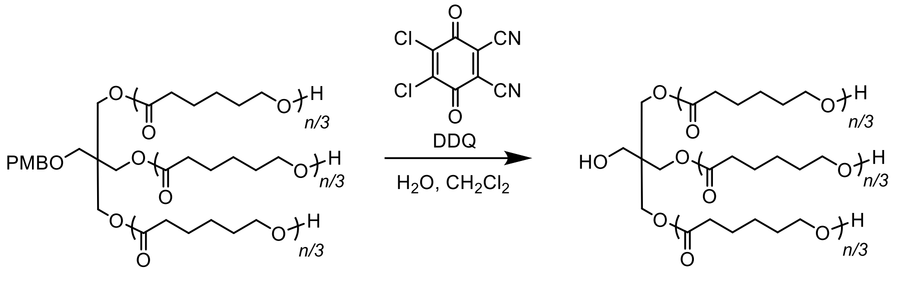

Method C was used for the deprotection reaction of PMBO-(PCL-OH)$_3$ ($M_{n,NMR}$ = 4,820 g mol^{-1}, 2.0 g, 415 μmol) with DDQ (188 mg, 830 μmol) in H_2O/CH_2Cl_2 (1.50 mL, v/v = 2/1) to give HO-(PCL-OH)$_3$ as white solid (1.17 g). Yield: 60.0%

$M_{n,NMR}$ = 5,350 g mol^{-1}, $M_{n,SEC}$ = 8,020 g mol^{-1}, $Đ$ = 1.08

^{1}H NMR (400 MHz, $CDCl_3$): δ (ppm) 4.31-3.78 (m, -OCO(CH_2)$_4$CH_2-), 3.65 (t, J = 6.40, -OCO(CH_2)$_3$CH_2OH), 3.61-3.44 (m, -CCH_2OH,) 2.56-2.09 (m, -OCOCH_2(CH_2)$_4$-), 1.95-1.52 (m, -OCOCH_2CH_2(CH_2)$_3$-, -OCO(CH_2)$_3$CH_2CH_2-), 1.47-1.27 (m, -OCO(CH_2)$_2$CH_2(CH_2)$_2$-).

Synthesis of P3-a

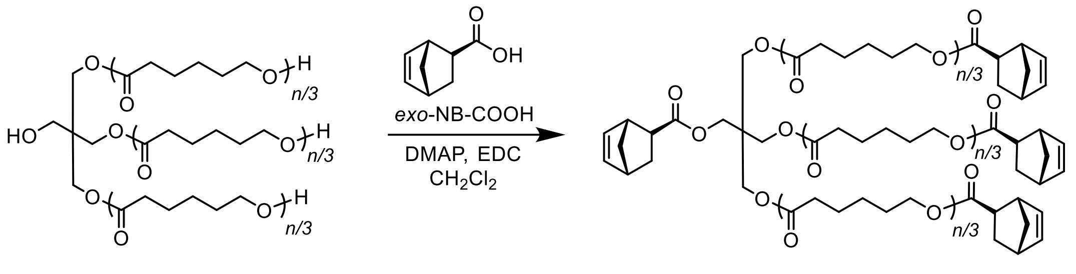

HO-(PCL-OH)3 P3

Method D was used for the condensation reaction of HO-(PCL-OH)3 ($M_{n,NMR}$ = 5,350 g mol^{-1}, 1.0 g, 185 μmol) with *exo*-NB-COOH (204 mg, 1.48 mmol), DMAP (274 mg, 2.24 mmol), and EDC (430 mg, 2.24 mmol) in CH_2Cl_2 (10 mL) to give **P3-a** as a white solid (576 mg). Yield: 60.1%

$M_{n,NMR}$ = 6,160 g mol^{-1}, $M_{n,SEC}$ = 8,650 g mol^{-1}, $Đ$ = 1.07

^{1}H NMR (400 MHz, $CDCl_3$): δ (ppm) 6.19-6.07 (m, -C*H*=C*H*- in norbornene ring), 4.18-4.00 (m, -OCO(CH_2)$_4$C*H*$_2$-), 3.05-2.98 (m, -C*H*-CH-CH_2O- in norbornene ring), 2.95-2.90 (s, -C*H*-CH-CH_2O- in norbornene ring), 2.43-2.25 (m, -OCOC*H*$_2$(CH_2)$_4$-), 1.78-1.56 (m, -OCOCH$_2$C*H*$_2$(CH_2)$_3$-, -OCO(CH_2)$_3$C*H*$_2$CH$_2$-), 1.48-1.31 (m, -OCO(CH_2)$_2$C*H*$_2$(CH_2)$_2$-).

Synthesis of MC3-a (trefoil-shaped PCL)

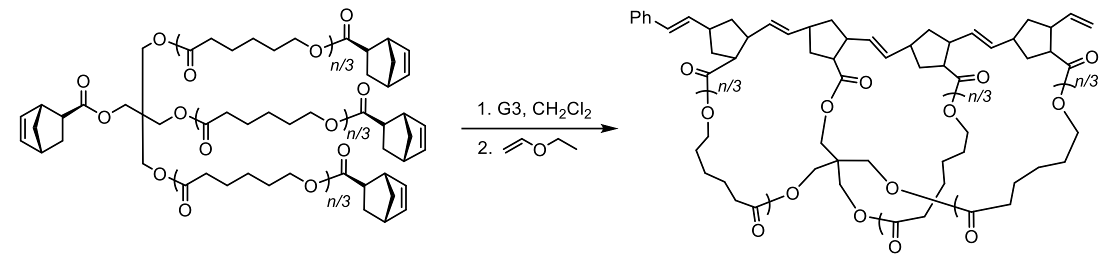

Method E was used for the ROMO of **P3-a** ($M_{n,NMR}$ = 6,160 g mol^{-1}, 30.0 mg, 4.92 µmol, 170 µM in CH_2Cl_2) with G3 (25.9 mg, 29.2 µmol) in CH_2Cl_2 (250 mL) to give **MC3-a** as a pale brown solid (27.7 mg). Yield: 90.0%

$M_{n,SEC}$ = 6,180 g mol^{-1}, $Đ$ = 1.09

^{1}H NMR (400 MHz, $CDCl_3$): δ(ppm) 6.52-1.73 (br, alkenyl of poly(norbornene) backbone), 4.32-3.80 (m, -OCO(CH_2)$_4$CH_2-), 2.42-2.14 (m, -OCOCH_2(CH_2)$_4$-), 1.79-1.49 (m, -OCOCH$_2$CH_2(CH_2)$_3$-, -OCO(CH_2)$_3$CH_2CH$_2$-), 1.45-1.07 (m, -OCO(CH_2)$_2$CH_2(CH_2)$_2$-).

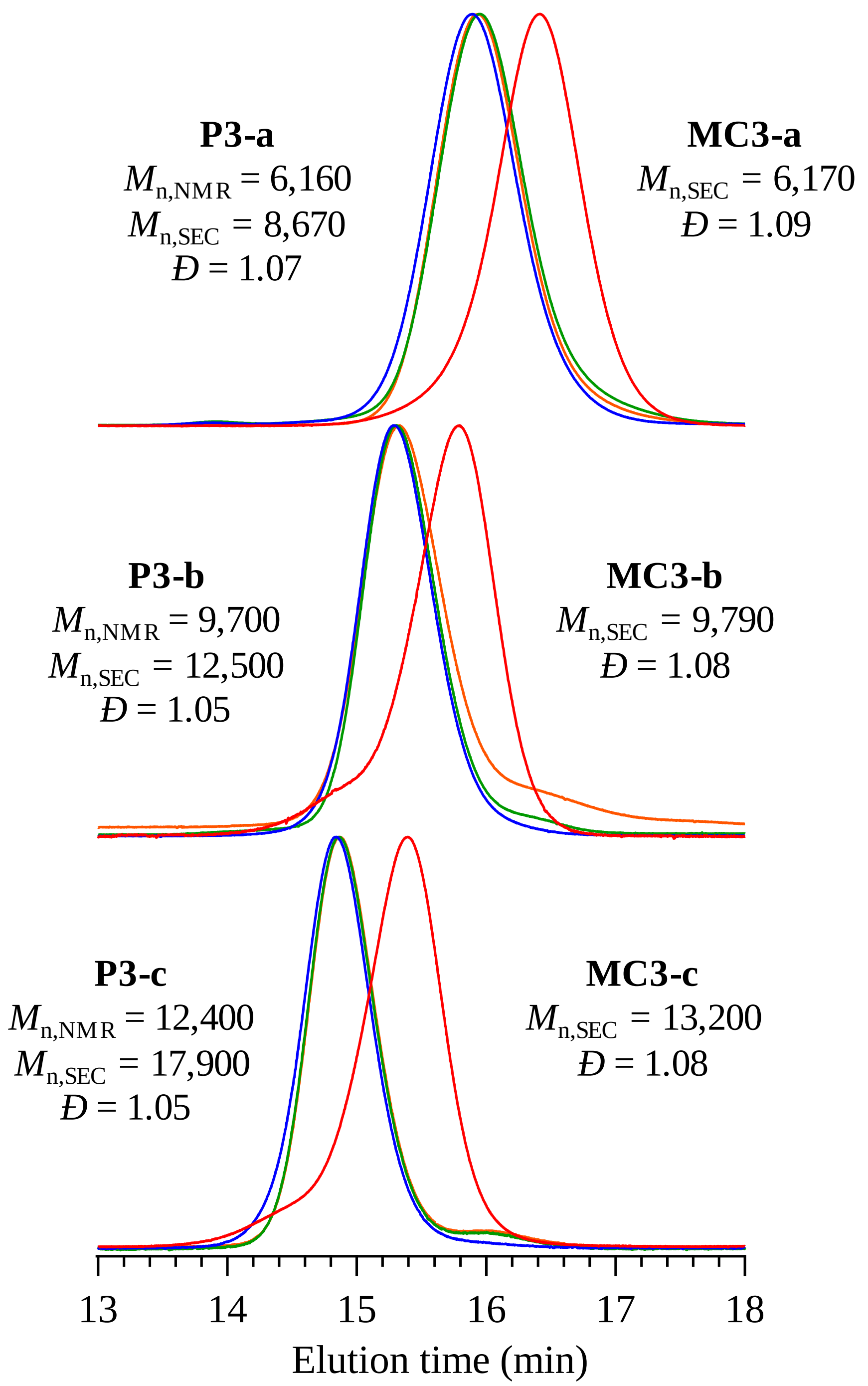

Supplementary Figure 4. SEC traces of PMBO-(PCL-OH)$_3$s (orange), HO-(PCL-OH)$_3$s (green), **P2**s (blue) and **MC3**s (red) with different molecular weight.

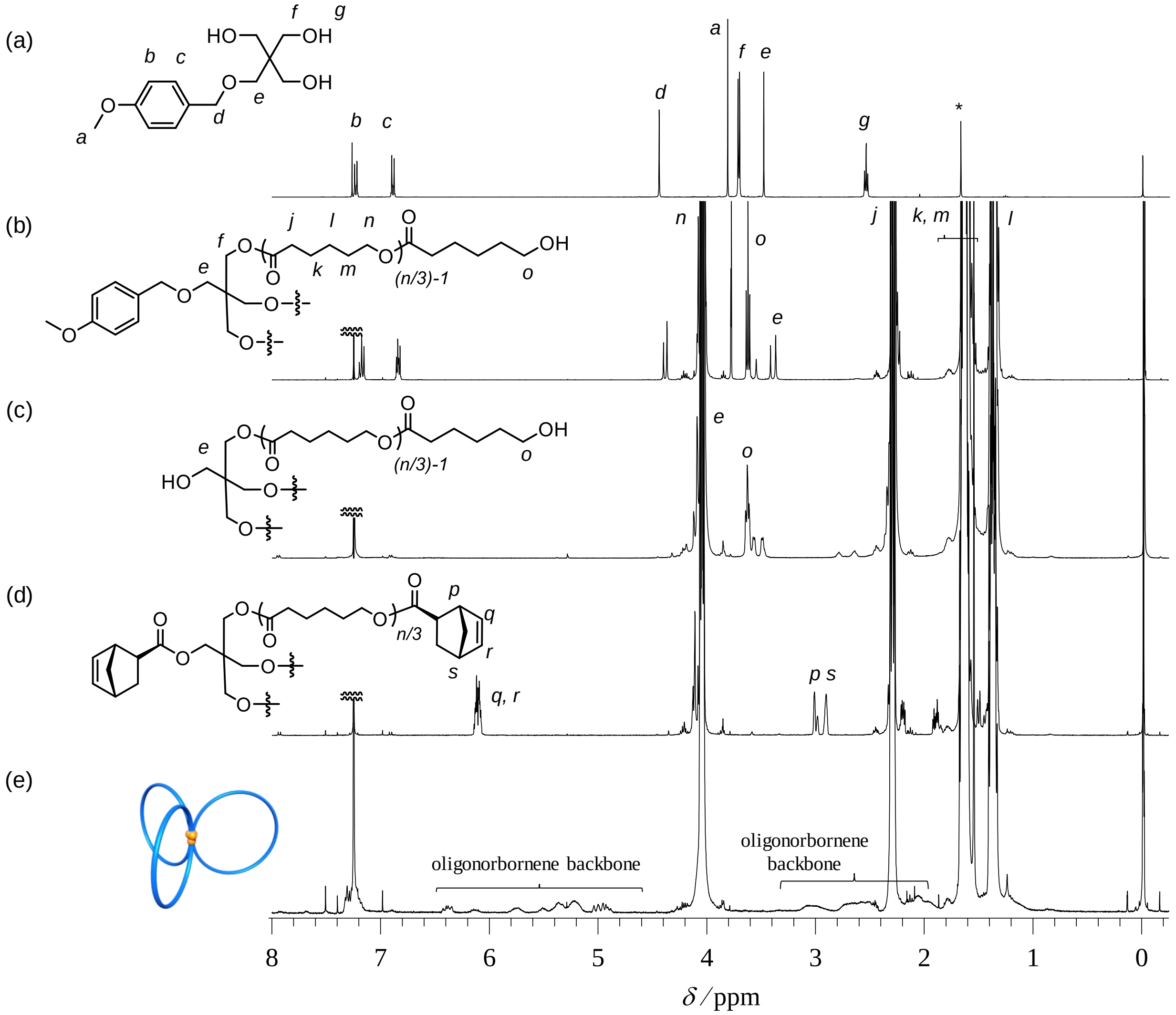

Supplementary Figure 5. ^{1}H NMR spectra of (a) **I2**, (b) PMBO-(PCL-OH)$_3$, (c) HO-(PCL-OH)$_3$, (d) **P3-a**, and (e) **MC3-a** in $CDCl_3$ (400 MHz).

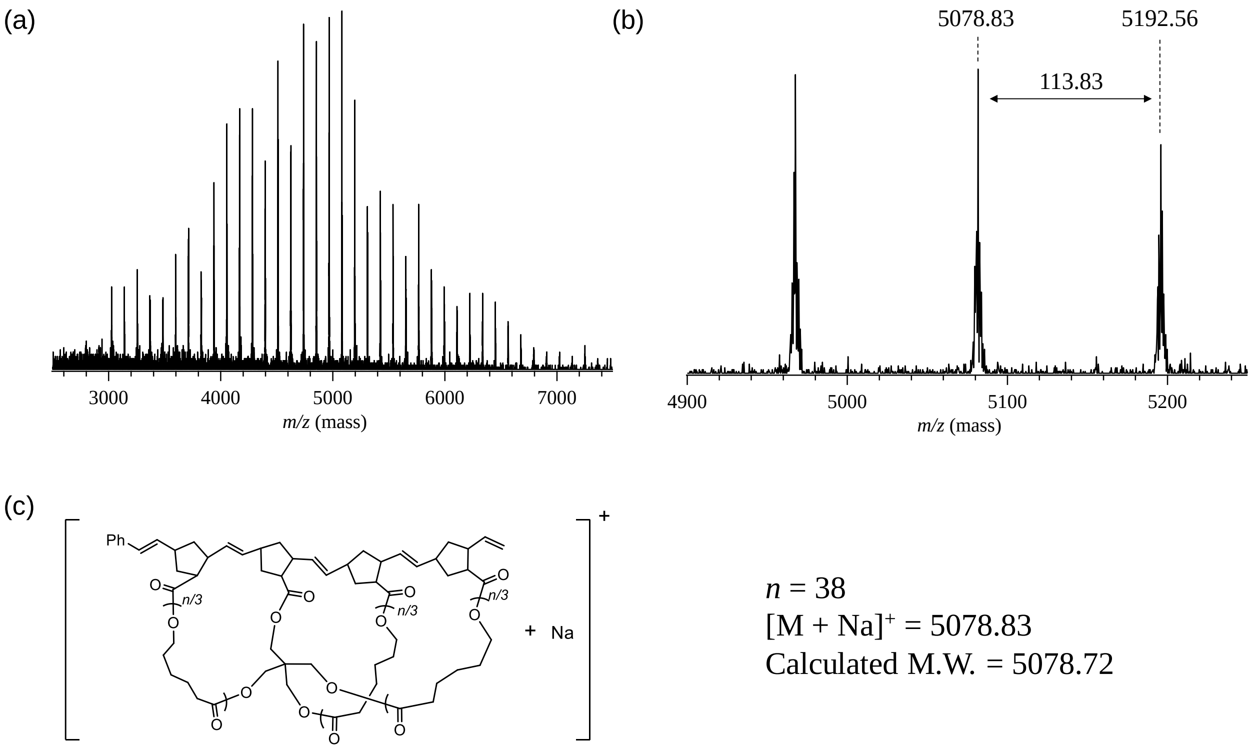

Supplementary Figure 6. MALDI-TOF MS analysis of **MC3-a**.

Supplementary Table 2. Molecular characterization of **MC3**s and their precursors

Sample	$M_{n,NMR}$ [a]	$M_{n,SEC}$ [b]	$M_{w,MALS}$ [c]	$Đ$ [b]	yield (%)	D_h [c] (nm)	$[\eta]$ [c] (mL g^{-1})	T_m [d] (°C)	X_{WAXD} [e] (%)
PMBO-(PCL-OH)$_3$-a	4,820	8,190	-	1.08	88.3	-	-	-	-
HO-(PCL-OH)$_3$-a	5,350	8,020	-	1.09	60.0	-	-	-	-
P3-a	6,160	8,670	5,330	1.07	60.1	4.4	14.3	42.6	39.3
MC3-a	-	6,170	4,820	1.09	90.0	3.6	6.9	42.6	31.7
PMBO-(PCL-OH)$_3$-b	8,830	11,200	-	1.09	89.1	-	-	-	-
HO-(PCL-OH)$_3$-b	10,700	12,100	-	1.06	72.8	-	-	-	-
P3-b	9,700	12,500	8,380	1.05	73.7	5.8	20.6	48.8	44.0
MC3-b	-	9,790	10,400	1.08	64.7	4.8	11.3	52.8	42.8
PMBO-(PCL-OH)$_3$-c	12,700	16,700	-	1.06	80.0	-	-	-	-
HO-(PCL-OH)$_3$-c	12,300	16,800	-	1.06	88.2	-	-	-	-
P3-c	12,400	17,900	10,300	1.05	77.5	6.8	24.9	50.9	46.3
MC3-c	-	13,200	12,100	1.08	91.2	5.6	13.2	54.4	45.4

[a] Determined by ^{1}H NMR. [b] Determined by SEC in THF using PSt standards. [c] Determined by SEC-MALS-Visco in THF. [d] Determined from a melting peak of the DSC curve. [e] Determined by WAXD at r.t..

Synthesis of PMBO-(PCL-OH)$_4$

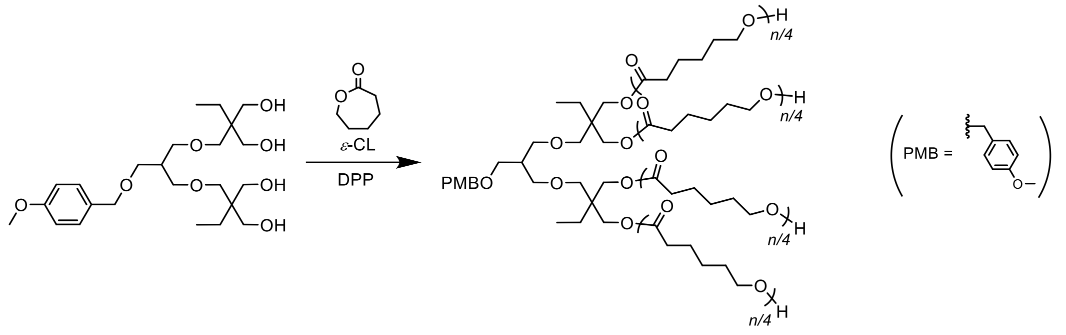

Method B was used for the polymerization of ε-CL (3.0 g, 26.3 mmol) with **I3** (241 mg, 525 μmol), and DPP (131 mg, 525 μmol) in toluene (20.2 mL) at r.t. for 3 h to give PMBO-(PCL-OH)$_3$ as white solid (1.66 g). Yield: 66.3%

$M_{n,NMR}$ = 5,490 g mol^{-1}, $M_{n,SEC}$ = 9,240 g mol^{-1}, $Đ$ = 1.04

^{1}H NMR (400 MHz, $CDCl_3$): δ (ppm) 7.23 (d, J = 8.70, aromatic), 6.87 (d, J = 8.20, aromatic), 4.39 (s, -PhCH_2O-), 4.13-4.00 (m, -OCO(CH_2)$_4$CH_2-), 3.97 (s, -CH_2OCO-), 3.80 (s, -OCH_3), 3.65 (t, J = 6.40, -CH_2OH), 3.44 (d, J = 5.90, -PhCH_2OCH_2-), 3.40 (d, J = 5.90, -CH_2OCH_2C(CH_3)-), 3.24 (s, -CH_2OCH_2C(CH_3)-), 2.39-2.20 (m, -OCOCH_2(CH_2)$_4$-), 1.71-1.54 (m, -OCOCH_2CH_2(CH_2)$_3$-, -OCO(CH_2)$_3$C$H_2$$CH_2$-), 1.49-1.31 (m, -OCO($CH_2$)$_2CH_2$($CH_2$)$_2$-) 0.95 (s, -C(C$H_3$)-).

Synthesis of HO-(PCL-OH)$_4$

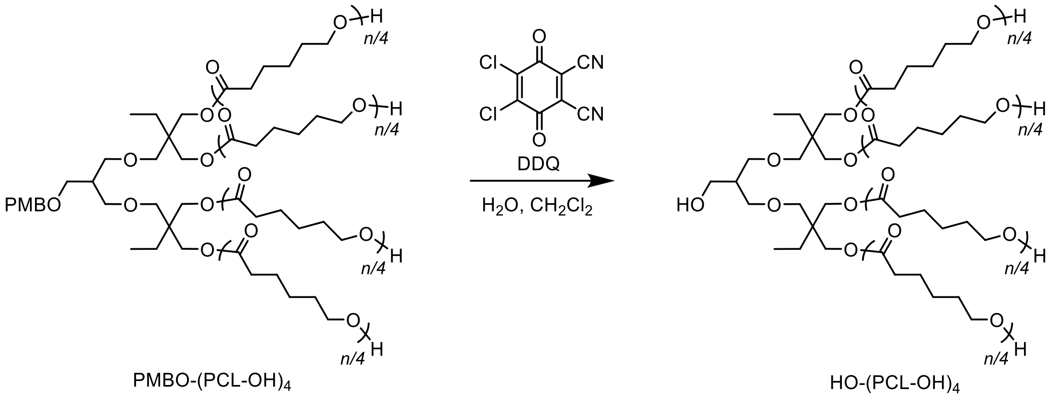

Method C was used for the deprotection reaction of PMBO-(PCL-OH)$_4$ ($M_{n,NMR}$ = 5,490 g mol^{-1}, 1.5 g, 273 μmol) with DDQ (124 mg, 546 μmol) in H_2O/CH_2Cl_2 (1.50 mL, v/v = 2/1) to give HO-(PCL-OH)$_4$ as white solid (691 mg). Yield: 47.3%

$M_{n,NMR}$ = 5,250 g mol^{-1}, $M_{n,SEC}$ = 9,110 g mol^{-1}, $Đ$ = 1.05,

^{1}H NMR (400 MHz, $CDCl_3$): δ (ppm) 4.12-4.02 (m, -OCO(CH_2)$_4$CH_2-), 3.99 (s, -CH_2OCO-), 3.65 (t, J = 6.40, -CH_2OH), 3.48-3.40 (m, -PhCH$_2$OCH_2-, -CH$_2$OCH_2C(CH$_3$)-), 3.26 (s, -CH_2OCH$_2$C(CH$_3$)-), 2.49-2.19 (m, -OCOCH_2(CH$_2$)$_4$-), 1.87-1.49 (m, -OCOCH$_2$CH_2(CH$_2$)$_3$-, -OCO(CH$_2$)$_3$CH_2CH$_2$-), 1.48-1.31 (m, -OCO(CH$_2$)$_2$CH_2(CH$_2$)$_2$-), 0.97 (s, -C(CH_3)-).

Synthesis of P4-a

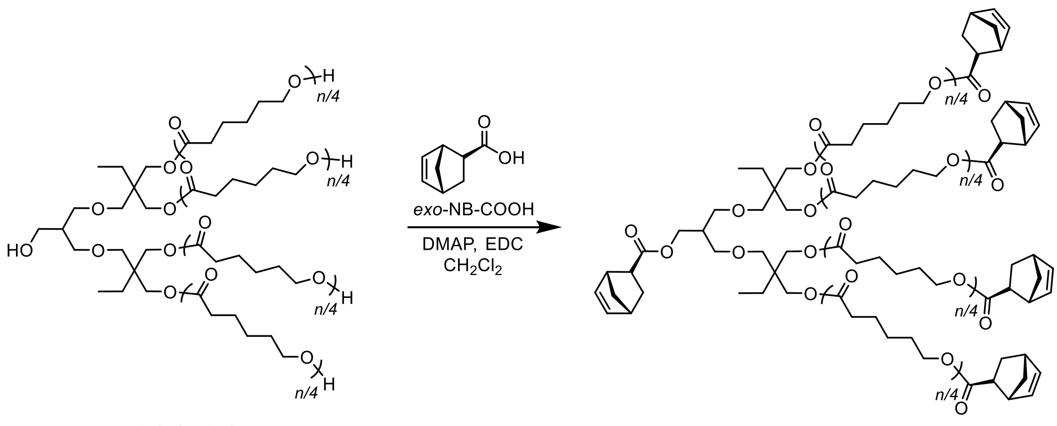

Method D was used for the condensation reaction of HO-(PCL-OH)3 ($M_{n,NMR}$ = 5,250 g mol^{-1}, 600 mg, 114 μmol) with *exo*-NB-COOH (156 mg, 1.14 mmol), DMAP (206 mg, 1.71 mmol), and EDC (329 mg, 1.71 mmol) in CH_2Cl_2 (6.0 mL) to give **P4-a** as a white solid (290 mg). Yield: 48.3%

$M_{n,NMR}$ = 6,620 g mol^{-1}, $M_{n,SEC}$ = 9,450 g mol^{-1}, $Đ$ = 1.04,

^{1}H NMR (400 MHz, $CDCl_3$): δ (ppm) 6.22-6.03 (m, -C*H*=C*H*- in norbornene ring), 4.16-4.01 (m, -OCO(CH_2)$_4$C*H*$_2$-), 3.98 (s, -C*H*$_2$OCO-), 3.48-3.36 (m, -PhCH$_2$OC*H*$_2$-), 3.26 (s, CH$_2$OC*H*$_2$C(CH$_3$)-), 3.06-3.00 (m, -C*H*-CH-CH$_2$O- in norbornene ring), 2.92 (s, -C*H*-CH-CH$_2$O- in norbornene ring), 2.40-2.25 (m, -OCOC*H*$_2$(CH$_2$)$_4$-), 1.77-1.54 (m, -OCOCH$_2$C*H*$_2$(CH$_2$)$_3$-, -OCO(CH$_2$)$_3$C*H*$_2$CH$_2$-), 1.46-1.27 (m, -OCO(CH$_2$)$_2$C*H*$_2$(CH$_2$)$_2$-), 0.97 (s, -C(C*H*$_3$)-).

Synthesis of MC4-a (quatrefoil-shaped PCL)

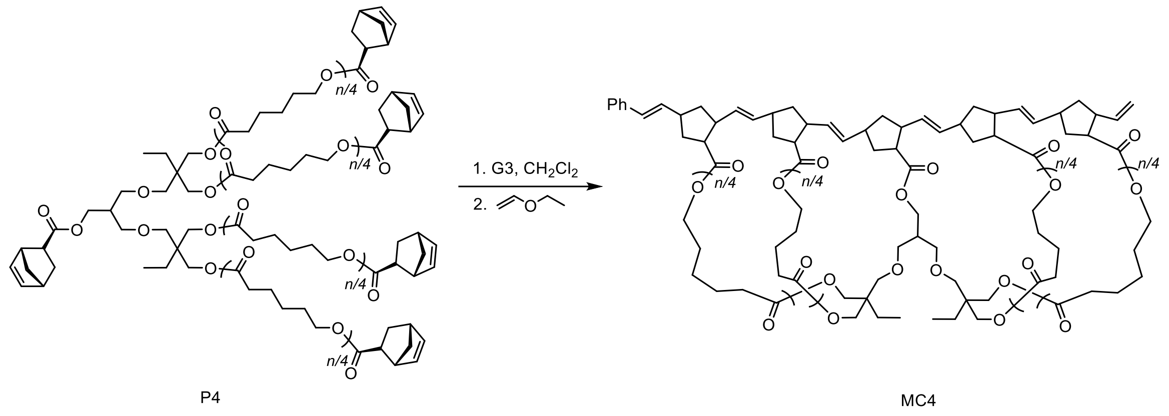

Method E was used for the ROMO of **P4-a** ($M_{n,NMR}$ = 6,620 g mol^{-1}, 30.0 mg, 4.55 μmol, 170 μM in CH_2Cl_2) with G3 (24.1 mg, 27.2 μmol) in CH_2Cl_2 (230 mL) to give **MC4-a** as a pale brown solid (27.4 mg). Yield: 89.7%

$M_{n,SEC}$ = 6,710 g mol^{-1}, $Đ$ = 1.07

^{1}H NMR (400 MHz, $CDCl_3$): δ(ppm) 6.61-2.38 (br, alkenyl of poly(norbornene) backbone), 4.25-3.80 (m, -OCO(CH_2)$_4$CH_2-), 2.41-2.12 (m, -OCOCH_2(CH_2)$_4$-), 1.89-1.47 (m, -OCOCH$_2$CH_2(CH_2)$_3$-, -OCO(CH_2)$_3$CH_2CH$_2$-), 1.48-1.15 (m, -OCO(CH_2)$_2$CH_2(CH_2)$_2$-), 1.01-0.57 (s, -C(CH_3)-).

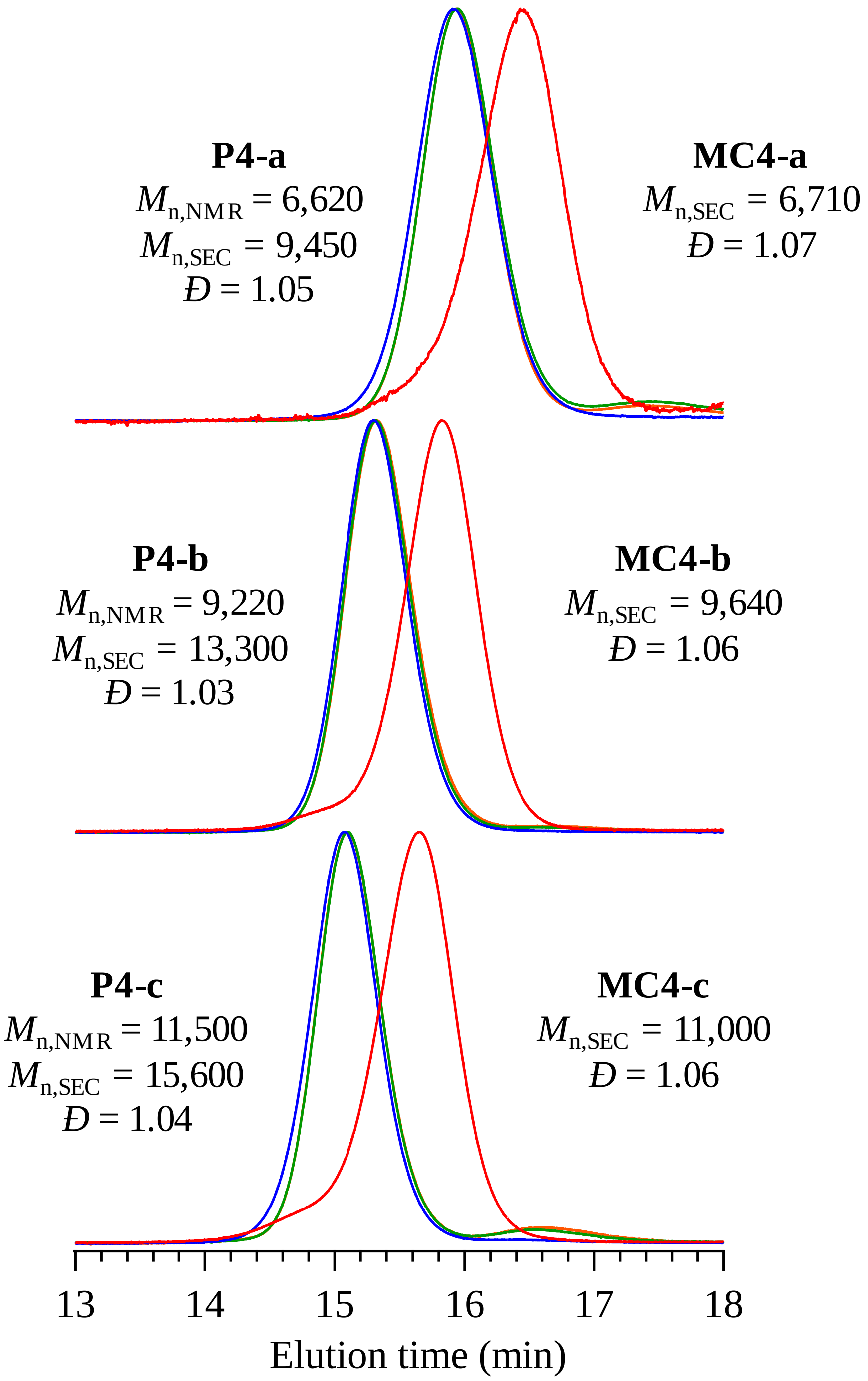

Supplementary Figure 7. SEC traces of PMBO-(PCL-OH)4s (orange), HO-(PCL-OH)4s (green), **P4**s (blue) and **MC4**s (red) with different molecular weight.

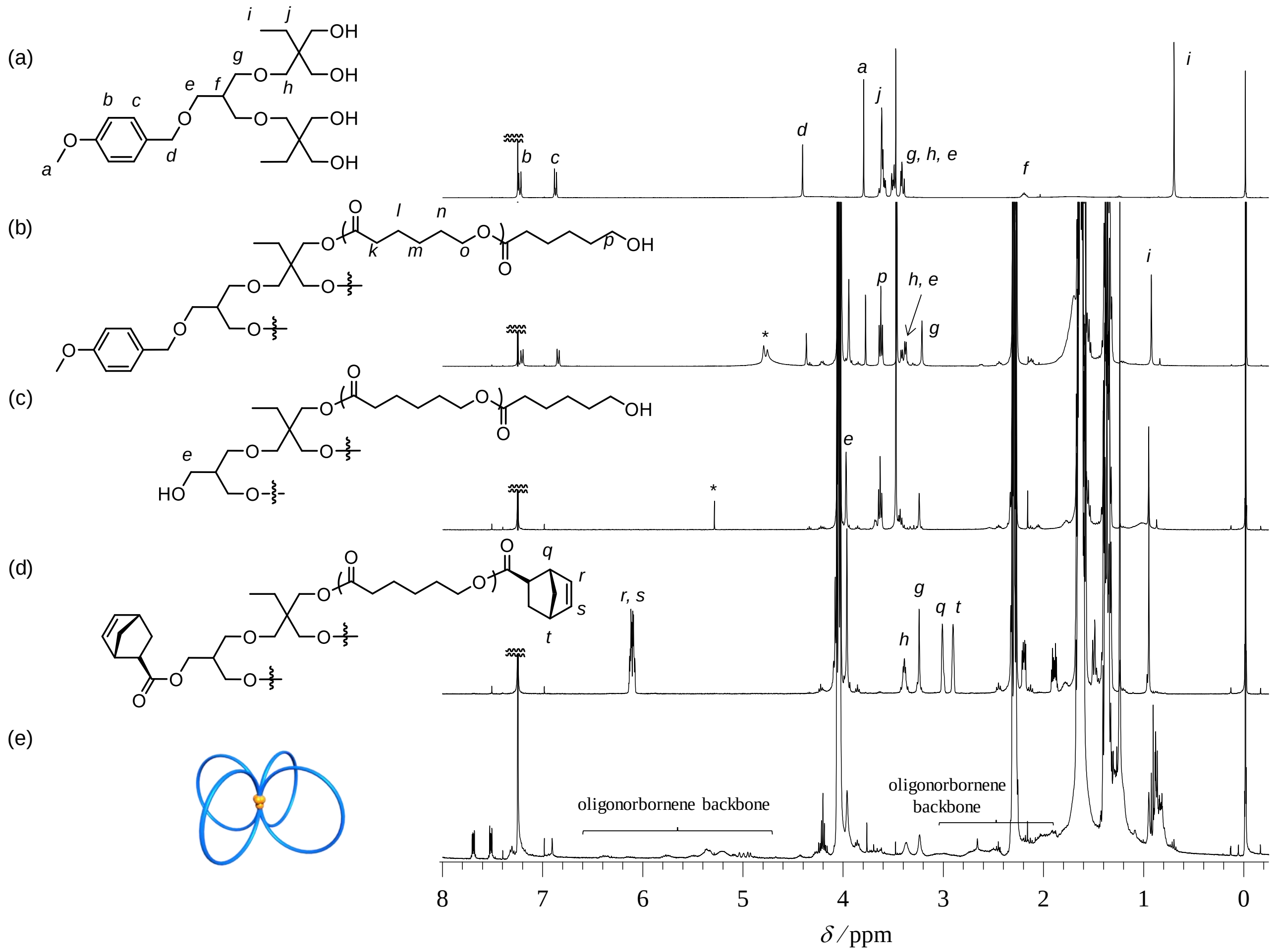

Supplementary Figure 8. ^{1}H NMR spectra of (a) **I3**, (b) PMBO-(PCL-OH)$_4$, (c) HO-(PCL-OH)$_4$, (d) **P4-a**, and (e) **MC4-a** in $CDCl_3$ (400 MHz).

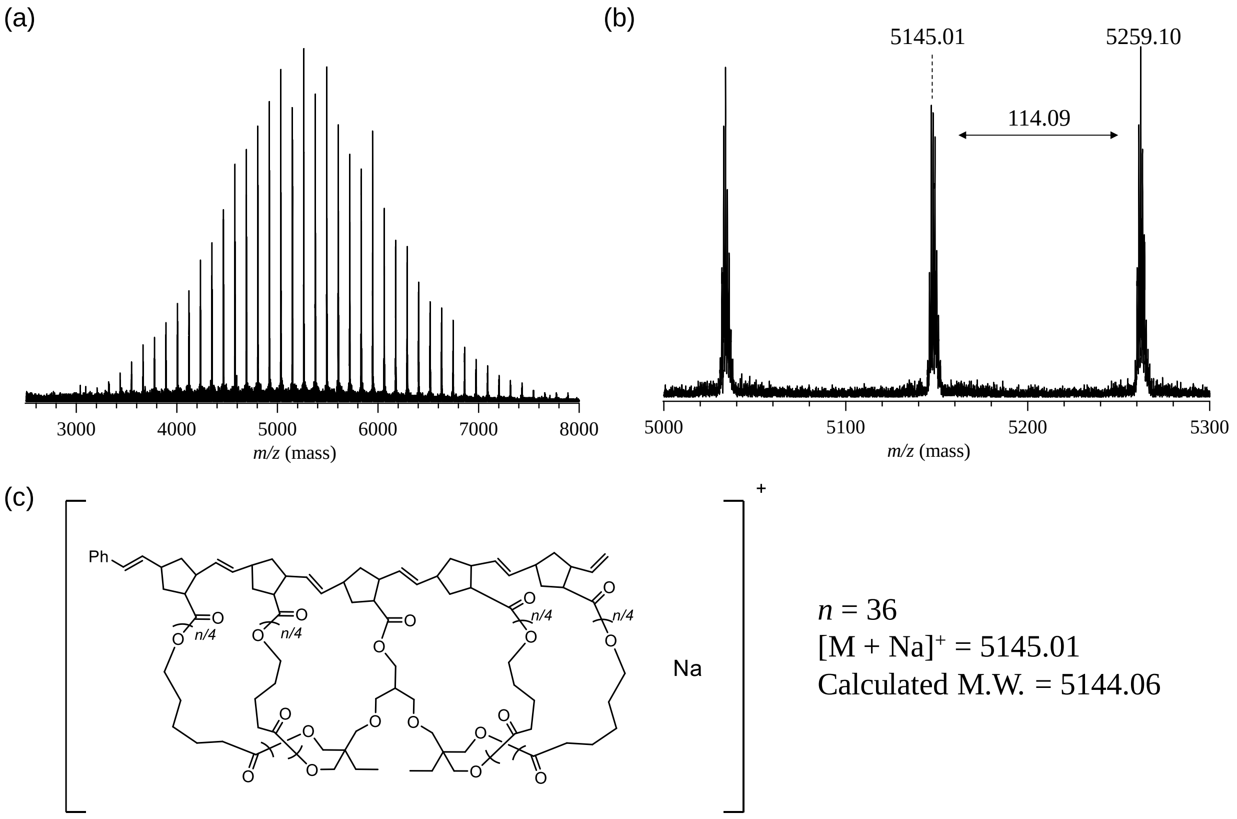

Supplementary Figure 9. MALDI-TOF MS analysis of **MC4-a**.

Supplementary Table 3. Molecular characterization of **MC4**s and their precursors

Sample	$M_{n,NMR}$ [a]	$M_{n,SEC}$ [b]	$M_{w,MALS}$ [c]	$Đ$ [b]	yield (%)	D_h [c] (nm)	$[\eta]$ [c] (mL g^{-1})	T_m [d] (°C)	X_{WAXD} [e] (%)
PMBO-(PCL-OH)$_4$-a	5,490	9,240	-	1.04	66.3	-	-	-	-
HO-(PCL-OH)$_4$-a	5,250	9,110	-	1.05	47.3	-	-	-	-
P4-a	6,620	9,450	6,380	1.05	48.3	4.6	13.6	38.0	34.4
MC4-a	-	6,710	6,900	1.07	89.7	4.0	6.9	29.3	23.6
PMBO-(PCL-OH)$_4$-b	9,930	12,900	-	1.04	76.0	-	-	-	-
HO-(PCL-OH)$_4$-b	8,800	13,000	-	1.03	73.4	-	-	-	-
P4-b	9,220	13,300	8,340	1.03	67.5	5.6	17.6	45.2	40.7
MC4-b	-	9,640	8,740	1.06	89.3	4.4	8.6	47.7	39.3
PMBO-(PCL-OH)$_4$-c	11,200	15,200	-	1.03	77.3	-	-	-	-
HO-(PCL-OH)$_4$-c	10,900	15,200	-	1.03	69.4	-	-	-	-
P4-b	11,500	15,600	10,000	1.04	64.1	6.0	19.9	47.0	43.4
MC4-b	-	11,000	10,400	1.06	85.3	5.0	9.8	50.8	44.4

[a] Determined by ^{1}H NMR. [b] Determined by SEC in THF using PSt standards. [c] Determined by SEC-MALS-Visco in THF. [d] Determined from a melting peak of the DSC curve. [e] Determined by WAXD at r.t..

Functionalization of *spiro*-multicyclic polymers

The syntheses of the α-/ω-hydroxyl-functionalized trefoil-shaped PCLs (namely, **MC3-OH$^{\alpha}$** and **MC3-OH$^{\omega}$**; M.W. = ca. 6000) were carried out using 4-(hydroxymethyl)styrene or (*Z*)-2-butene-1,4-diol in one pot, respectively. The introduced hydroxyl group can be a versatile scaffold for post-polymerization modification, allowing access to the functionalized structures and higher-ordered architectures. Despite the complicated structure of the multicyclic polymer, both of end-functionalization approaches was readily achieved in high purity, as confirmed by SEC, ^{1}H NMR, and MALDI-TOF MS analyses (see Supplementary Figures 10-14). The synthetic procedures are as follows:

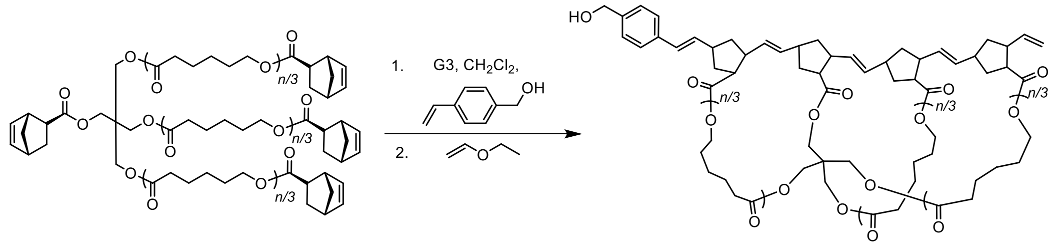

P3-a MC3-OH$^{\alpha}$

In a three-necked flask, G3 (12.9 mg, 14.6 μmol) and 4-(hydroxymethyl)styrene (22.2 mg, 165 μmol) was added to dry-CH_2Cl_2 (122 mL), and the solution was stirred for 1 h with Ar bubbling. Then, a solution of **P3-a** ($M_{n,NMR}$ = 6,160 g mol^{-1}, $M_{n,SEC}$ = 8,670, $Đ$ = 1.05, 15.0 mg, 2.44 μmol, 170 μM in CH_2Cl_2) was added dropwise to the G3 solution through the additional funnel over 20 min. After 5 min, the reaction was quenched by the addition of excess amount of ethyl vinyl ether. The metal residue and excess 4-(hydroxymethyl)styrene in the crude product were removed by preparative SEC (solvent, $CHCl_3$) to give **MC3-OH$^{\alpha}$** as a pale brown solid (14.1 mg). Yield: 91.6%

$M_{n,SEC}$ = 6,400, $Đ$ = 1.12

^{1}H NMR (400 MHz, $CDCl_3$): δ (ppm) 6.51-2.40 (br, alkenyl of poly(norbornene) backbone), 4.67 (s, -CH_2OH), 4.38-3.81 (m, -OCO$(CH_2)_4$CH_2-), 2.41-2.14 (m, -OCOC$H_2$$(CH_2)_4$-), 1.76-1.46 (m, -OCO$CH_2CH_2$$(CH_2)_3$-, -OCO$(CH_2)_3CH_2$$CH_2$-), 1.44-0.97 (m, -OCO$(CH_2)_2CH_2$$(CH_2)_2$-).

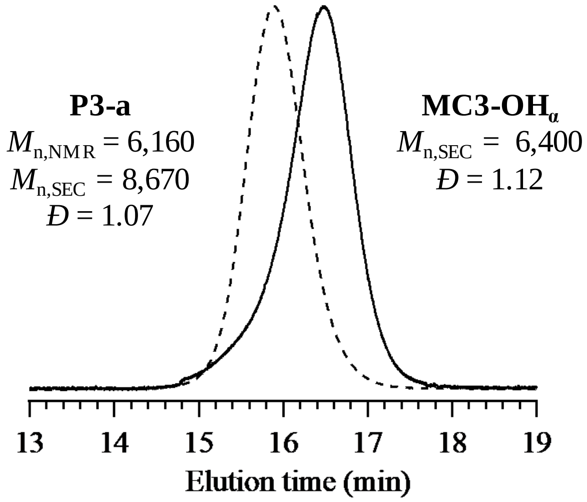

Supplementary Figure 10. SEC traces of **P3-a** (dotted line), **MC3-OH^α** (solid line).

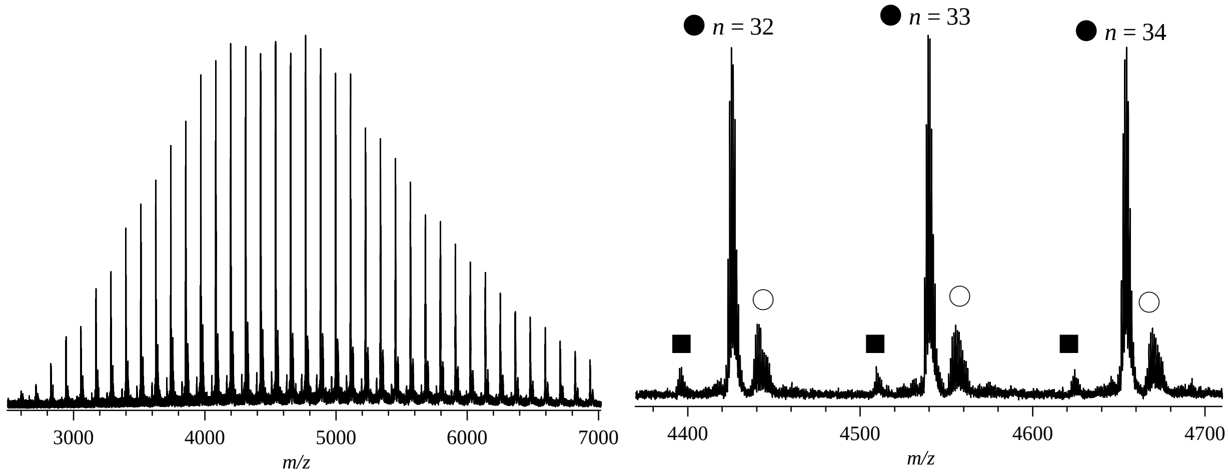

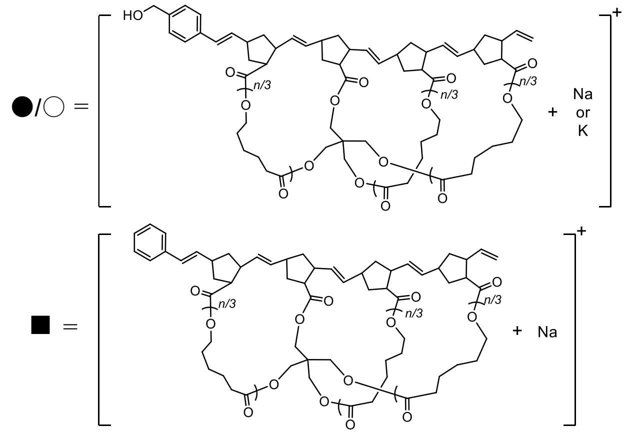

Calculated and observed M.W. of $[M + Na]^+$

	n = 32	n = 33	n = 34
Calcd.	4423.54	4537.61	4651.68
Obs.	4423.35	4537.41	4651.51

Supplementary Figure 11. MALDI-TOF MS analysis of **MC3-OH^α**.

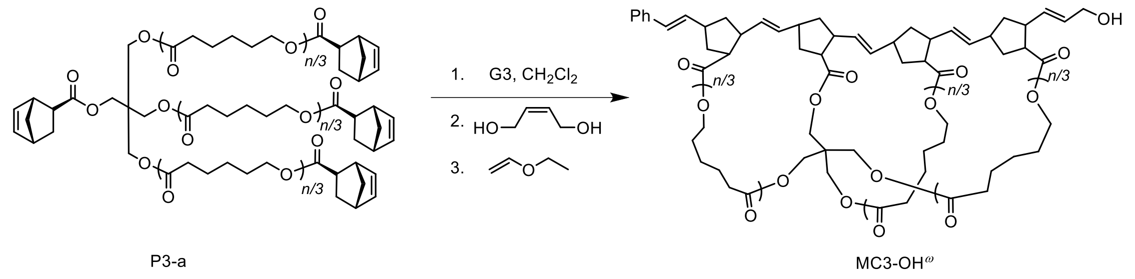

In a three-necked flask, G3 (8.6 mg, 9.67 μmol) was added to a three-necked flask and dissolved in dry-CH_2Cl_2 (81.0 mL). Then, a solution of **P3-a** ($M_{n,NMR}$ = 6,160 g mol^{-1}, $M_{n,SEC}$ = 8,670, $Đ$ = 1.05, 10.0 mg, 1.62 μmol, 170 μM in CH_2Cl_2) was added dropwise to the G3 solution through the additional funnel over 30 min. After 5 min, *cis*-2-butene-1,4-diol (18.2 mg, 206 μmol) was added to the reaction mixture and the solution was stirred for 1.5 h. The reaction was quenched by the addition of excess amount of ethyl vinyl ether. The metal residue and excess (*Z*)-2-butene-1,4-diol in the crude product were removed by preparative SEC (solvent, $CHCl_3$) to give **MC3-OH$^{\omega}$** as a pale brown solid (10.2 mg). Yield: 100%

$M_{n,SEC}$ = 6,290, $Đ$ = 1.13

^{1}H NMR (400 MHz, $CDCl_3$): δ(ppm) 6.49-2.41 (br, alkenyl of poly(norbornene) backbone), 4.33-3.90 (m, -OCO(CH_2)$_4$C*H*$_2$-, -C*H*$_2$OH), 2.39-2.21 (m, -OCOC*H*$_2$(CH_2)$_4$-), 1.74-1.48 (m, -OCOCH$_2$C*H*$_2$(CH_2)$_3$-, -OCO(CH_2)$_3$C*H*$_2$CH$_2$-), 1.45-1.16 (m, -OCO(CH_2)$_2$C*H*$_2$(CH_2)$_2$-).

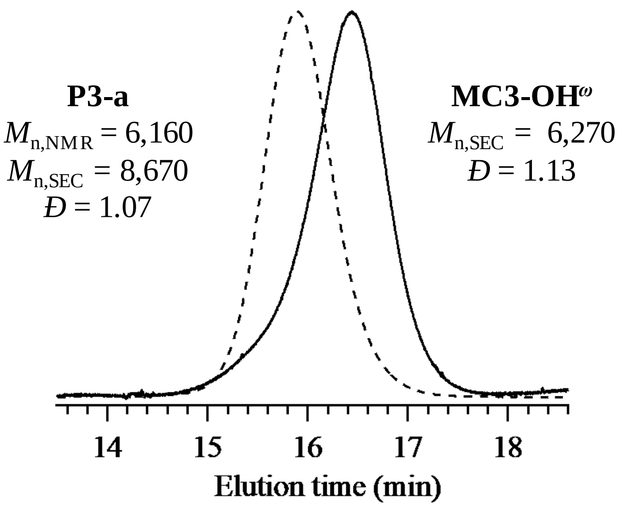

Supplementary Figure 12. SEC traces of **P3-a** (dotted line), **MC3-OH$^{\omega}$** (solid line).

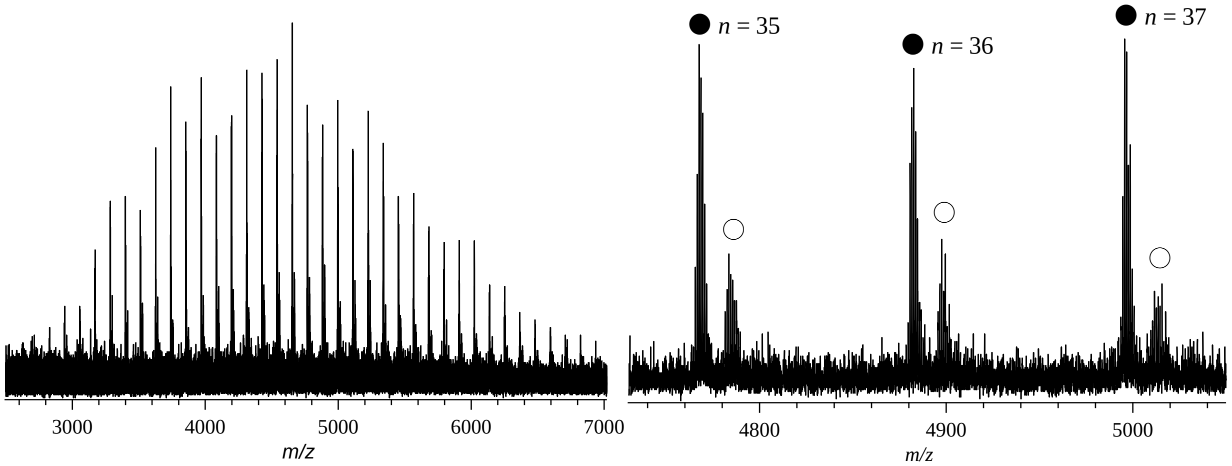

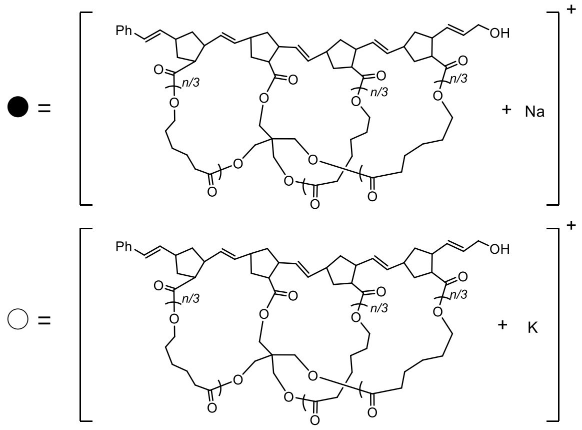

Calculated and observed M.W. of [M + Na]$^+$

	n = 35	n = 36	n = 37
Calcd.	4765.75	4882.82	4993.89
Obs.	4765.56	4882.63	4993.66

Supplementary Figure 13. MALDI-TOF MS analysis of **MC3-OH$^{\omega}$**.

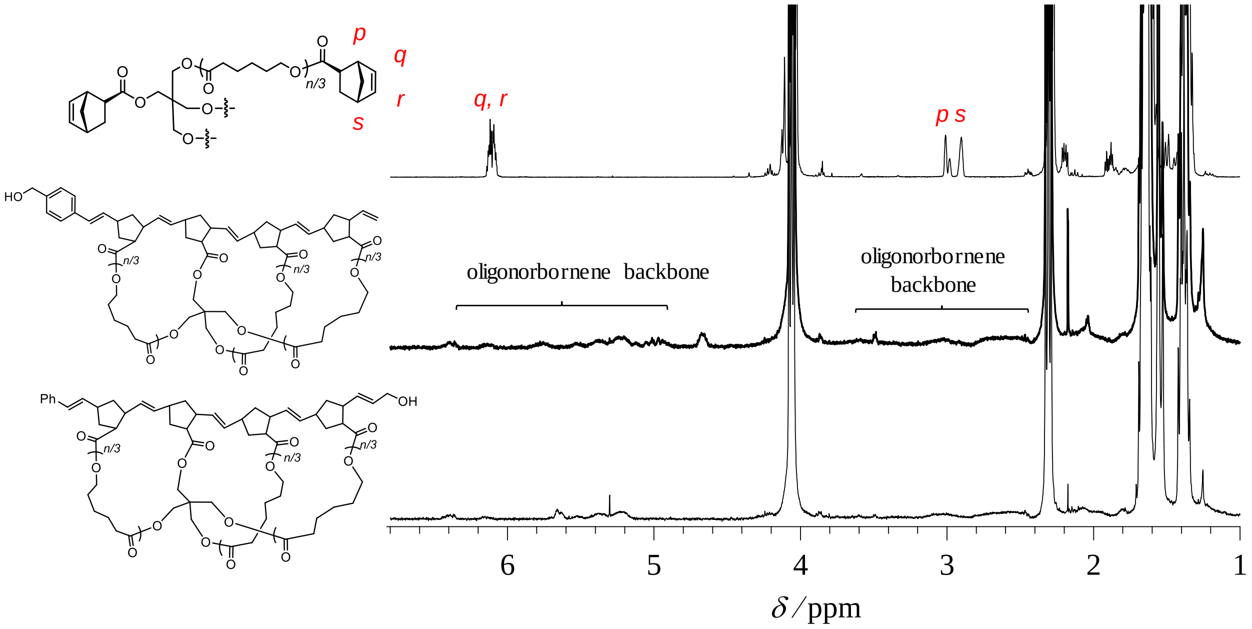

Supplementary Figure 14. ^{1}H NMR spectra of **P3-a** (upper), (b) **MC3-OH**$^{\alpha}$ (middle), and **MC3-OH**$^{\omega}$ (lower) in $CDCl_3$ (400 MHz).

Synthesis of *spiro*-multicyclic polylactide and polyether

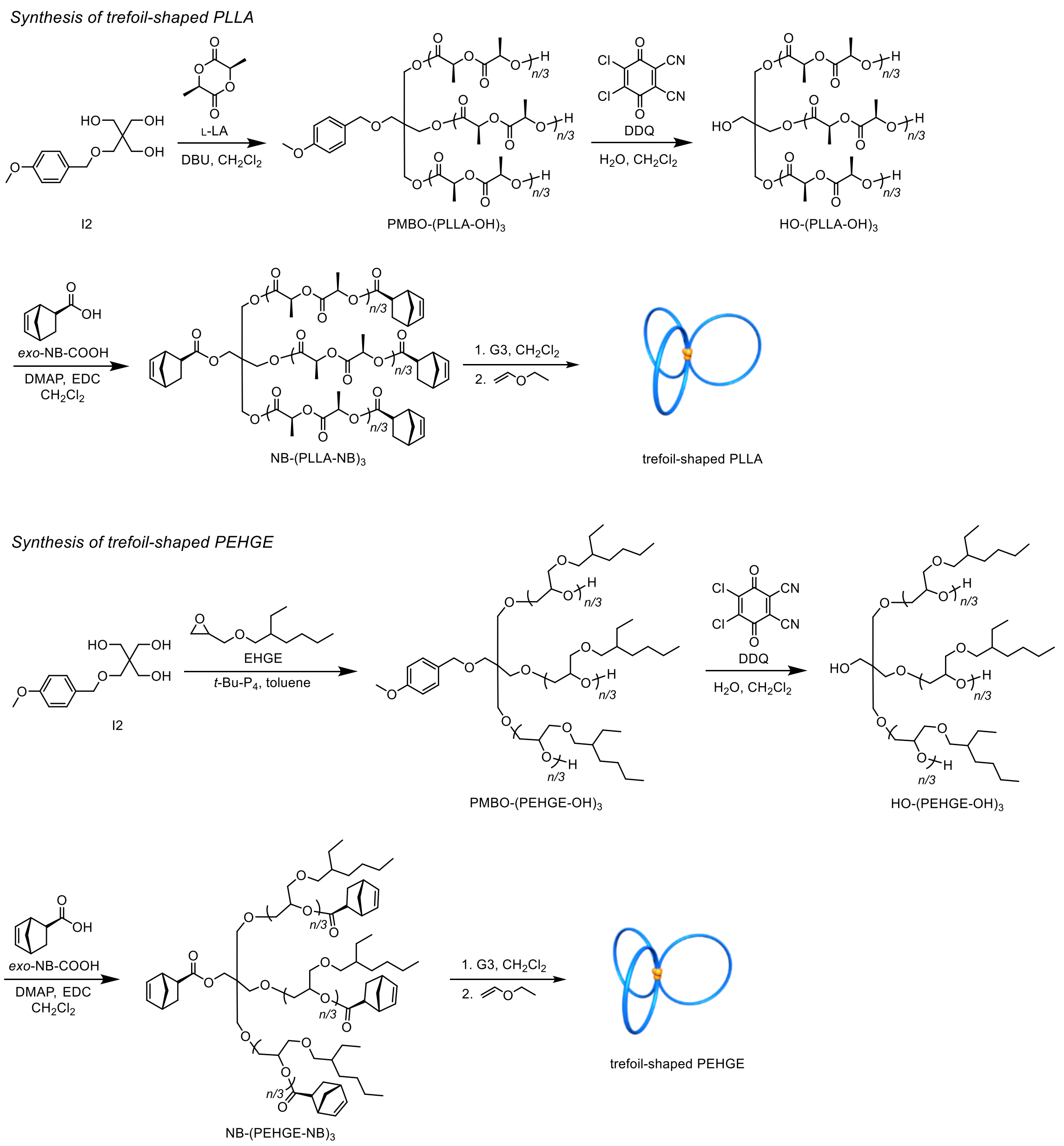

Supplementary Figure 15. Synthetic pathway for *spiro*-multicyclic PLLA and PEHGE.

Synthesis of PMBO-(PLLA-OH)$_3$

In a glovebox, **I3** (53.3 mg, 208 µmol) and L-LA (1.50 g, 10.4 mmol) were dissolved in dry-CH_2Cl_2 (21.0 mL). DBU (15.8 µL, 104 µmol) was then added to the CH_2Cl_2 solution to initiate the polymerization. After 7 min, the polymerization was quenched by the addition of excess amount of benzoic acid. The mixture was purified by reprecipitation from the CH_2Cl_2 solution to cold MeOH to give **PMBO-(PLLA-OH)$_3$** as a white solid (1.13 g). Yield: 86.9%

$M_{n,NMR}$ = 7,140 g mol^{-1}, $M_{n,SEC}$ = 10,800 g mol^{-1}, $Đ$ = 1.04

^{1}H NMR (400 MHz, $CDCl_3$): δ(ppm) 7.18 (d, J = 8.2, aromatic), 6.86 (d, J = 8.7, aromatic), 5.38-5.00 (m, methine of PLLA backbone), 4.47-4.28 (m, -PhCH_2O-, -CH(CH_3)OH), 4.16 (q, J = 11.4, -C(CH_2O-)$_3$), 3.80 (s, -OCH_3), 3.34 (s, -CH_2C(CH_2O-)$_3$), 2.66 (m, -OH), 1.86-1.34 (m, methyl of PLLA backbone).

Synthesis of HO-(PLLA-OH)$_3$

Method C was used for the deprotection reaction of PMBO-(PLLA-OH)$_3$ ($M_{n,NMR}$ = 7,140 g mol^{-1}, 1.02 g, 143 µmol) with DDQ (65.5 mg, 289 µmol) in H_2O/CH_2Cl_2 (15 mL, v/v = 1/2) for 24 h. After the purification, the same procedure was performed again because the quantitative reaction was not found by ^{1}H NMR analysis of the product. After 24 h, the polymer crude was purified by the reprecipitation twice from CH_2Cl_2 to cold methanol to give **HO-(PLLA-OH)$_3$** as white solid (443 mg). Yield: 44.3%

$M_{n,NMR}$ = 7,150 g mol^{-1}, $M_{n,SEC}$ = 10,900 g mol^{-1}, $Đ$ = 1.04

^{1}H NMR (400 MHz, $CDCl_3$): δ (ppm) 5.40-5.01 (m, methine of PLLA backbone), 4.34 (m, methine of ω-chain end lactyl unit), 4.15 (s, -C(C*H*$_2$O-)$_3$), 3.49 (ddd, J = 32.0, 11.9, 6.6 Hz, -CC*H*$_2$OH), 2.75-2.59 (m, -CHCH$_3$O*H*), 1.81-1.35 (m, methyl of PLLA backbone).

Synthesis of NB-(PLLA-NB)$_3$

Method D was used for the condensation reaction of HO-(PLLA-OH)$_3$ ($M_{n,NMR}$ = 7,150 g mol^{-1}, 403 mg, 56.4 μmol) with *exo*-NB-COOH (64.5 mg, 467 mmol), DMAP (82.7 mg, 677 mmol), and EDC (135 mg, 706 mmol) in CH_2Cl_2 (4.0 mL) to give **NB-(PLLA-NB)$_3$** as a white solid (293 mg). Yield: 67.5%

$M_{n,NMR}$ = 8,050 g mol^{-1}, $M_{n,SEC}$ = 11,400 g mol^{-1}, $Đ$ = 1.03

^{1}H NMR (400 MHz, $CDCl_3$): δ (ppm) 6.23-6.02 (m, -C*H*=C*H*- in norbornene ring), 5.41-4.91 (m, methine of PLLA backbone), 4.29-3.99 (m, -C*H*$_2$C(CH$_2$O-)$_3$, -C(C*H*$_2$O-)$_3$), 3.26-2.96 (m, -C*H*CHCO- in norbornene ring), 2.92 (s, -C*H*CH$_2$CHCO- in norbornene ring), 2.36-2.15 (m, -C*H*CO- in norbornene ring), 1.97 (m, *exo*-H of -C*H*$_2$- in norbornene ring), 1.74 (m, *endo*-H of -C*H*$_2$- in norbornene ring), 1.73-1.23 (m, methyl of PLLA backbone, bridge head -C*H*2- in norbornene ring).

Synthesis of trefoil-shaped PLLA

Method E was used for the ROMO of NB-(PLLA-NB)$_3$ ($M_{n,NMR}$ = 8,050 g mol^{-1}, 15.0 mg, 1.86 μmol, 170 μM in CH_2Cl_2) with G3 (9.9 mg, 11.2 μmol) in CH_2Cl_2 (93.0 mL) to give trefoil-shaped PLLA as a white solid (13.9 mg). Yield: 91.4%

$M_{n,SEC}$ = 7,700 g mol^{-1}, $Đ$ = 1.09

^{1}H NMR (400 MHz, $CDCl_3$): δ (ppm) 6.47-1.34 (br, alkenyl of poly(norbornene) backbone), 5.43-4.77 (m, m, methine of PLLA backbone), 1.34-1.81 (m, methyl of PLLA backbone).

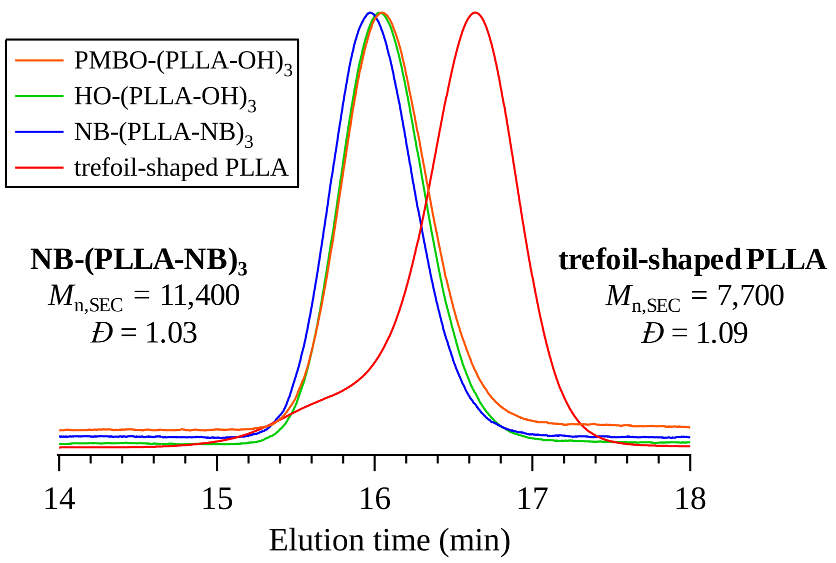

Supplementary Figure 16. SEC traces of PMBO-(PLLA-OH)$_3$ (orange), HO-(PLLA-OH)$_3$ (green), NB-(PLLA-NB)$_3$ (blue) and trefoil-shaped PLLA (red). The amount of shoulder peak in trefoil-shaped PLLA was calculated to be ca. 17.0% by peak deconvolution.

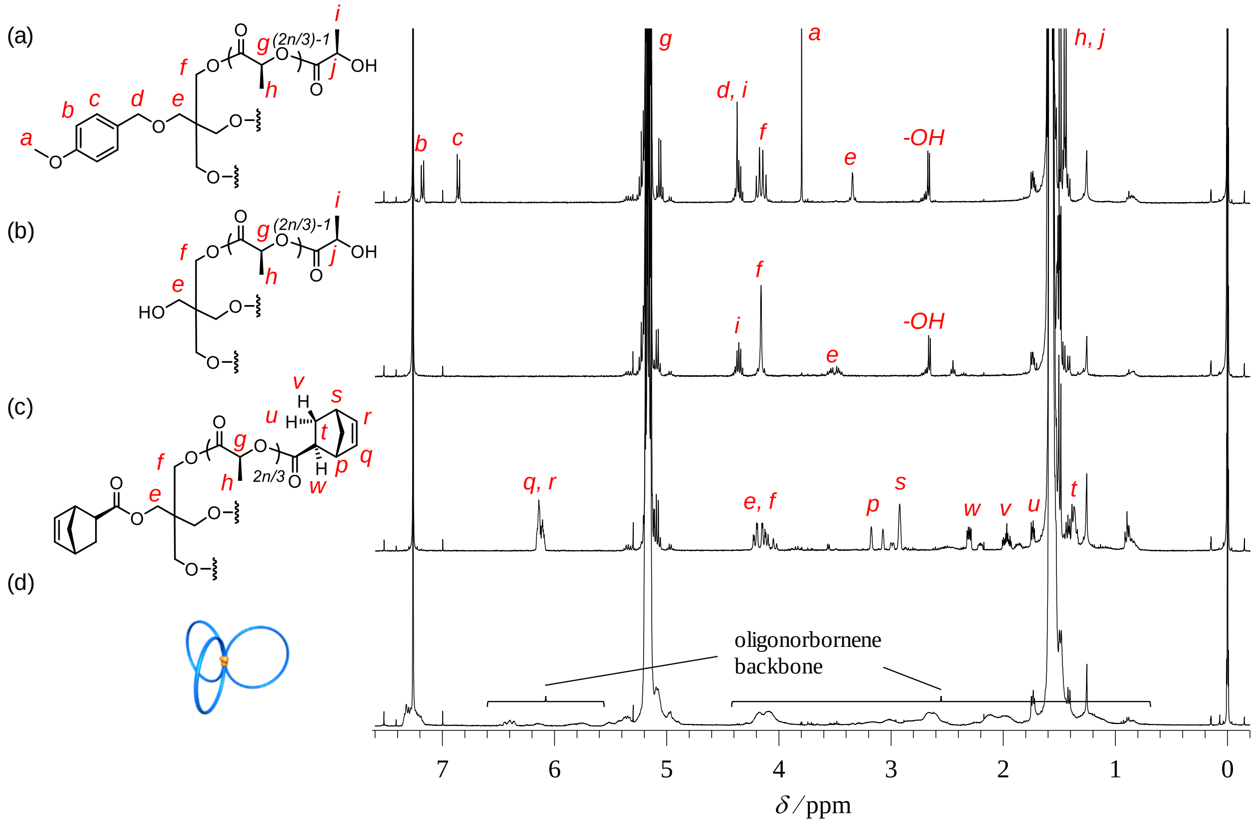

Supplementary Figure 17. ^{1}H NMR spectra of (a) PMBO-(PLLA-OH)$_3$, (b) HO-(PLLA-OH)$_3$, (c) NB-(PLLA-NB)$_3$, and (d) trefoil-shaped PLLA in $CDCl_3$ (400 MHz).

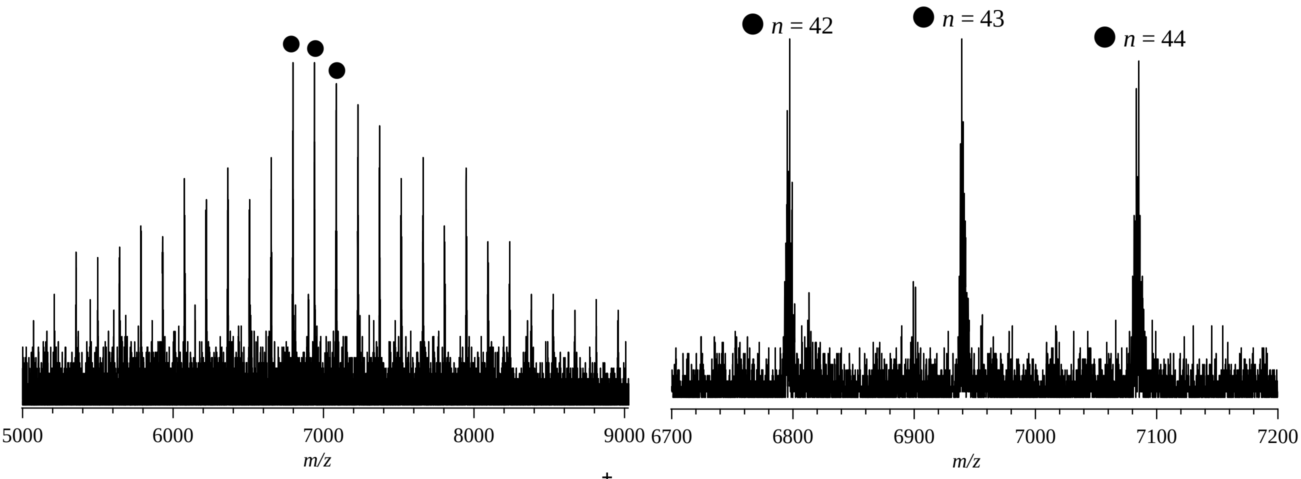

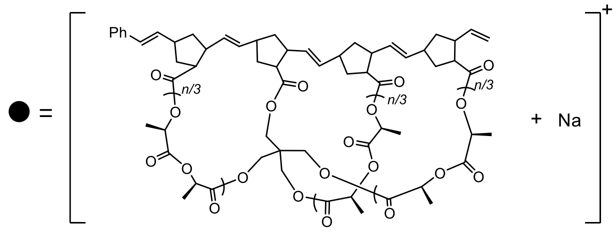

Calculated and observed M.W. of [M + Na]$^+$

	n = 42	*n* = 43	*n* = 44
Calcd.	6793.04	6937.08	7081.12
Obs.	6793.19	6937.27	7081.34

Supplementary Figure 18. MALDI-TOF MS analysis of trefoil-shaped PLLA.

Synthesis of PMBO-(PEHGE-OH)$_3$

In a glovebox, **I3** (28.3 mg, 110 μmol) and EHGE (0.80 mL, 3.87 mmol) were dissolved in dry-toluene (1.3 mL). *t*-Bu-P4 (138 μL of ~0.8 mol L^{-1} solution in *n*-hexane, 110 μmol) was then added to the toluene solution to initiate the polymerization. After 49 h, the polymerization was quenched by the addition of excess amount of benzoic acid. The product was purified by passing by a pad of alumina using dry-THF to give **PMBO-(PEHGE-OH)$_3$** as a colorless viscous liquid (472 mg). Yield: 63.0%

$M_{n,NMR}$ = 6,560 g mol^{-1}, $M_{n,SEC}$ = 7,170 g mol^{-1}, $Đ$ = 1.04

^{1}H NMR (400 MHz, $CDCl_3$): δ (ppm) 7.20 (d, J = 8.5, aromatic), 6.84 (d, J = 8.5, aromatic), 4.37 (s, -PhCH_2O-), 3.92 (m, -OH), 3.78 (s, CH_3O-) 3.66-3.57 (m, -CH_2CH(CH_2-)O-), 3.57-3.35 (-CH_2CH(CH_2-)O-, -CH_2CH(CH_2-)O-, -C(CH_2O-)$_3$), 3.35-3.22 (m, -CH_2CH(CH_2OCH_2-)O-), 1.55-1.43 (m, methine of EHGE side chain), 1.42-1.19 (m, methylene of EHGE side chain), 0.99-0.76 (m, methyl of EHGE side chain).

Synthesis of HO-(PEHGE-OH)$_3$

Method C was used for the deprotection reaction of PMBO-(PEHGE-OH)$_3$ ($M_{n,NMR}$ = 6,540 g mol^{-1}, 428 mg, 65.4 μmol) with DDQ (30.0 mg, 132 μmol) in H_2O/CH_2Cl_2 (6.4 mL, v/v = 1/2) for 24 h. After 24 h, the reaction mixture was dried by Na_2SO_4 and the product was purified by passing by a pad of alumina using dry-THF to give **HO-(PEHGE-OH)$_3$** as a colorless viscous liquid (233 mg). Yield: 55.6%

$M_{n,NMR}$ = 6,910 g mol^{-1}, $M_{n,SEC}$ = 7,390 g mol^{-1}, $Đ$ = 1.03

^{1}H NMR (400 MHz, $CDCl_3$): δ (ppm) 3.90 (m, -O*H*), 3.67-3.57 (m, -$CH_2CH(C\mathit{H}_2$-)O-), 3.57-3.37 (-C*H*$_2$CH(CH_2-)O-, -CH_2C*H*(CH_2-)O-, -C(C*H*$_2$O-)$_3$), 3.36-3.20 (m, -$CH_2CH(CH_2OC\mathit{H}_2$-)O-), 1.54-1.43 (m, methine of EHGE side chain), 1.42-1.18 (m, methylene of EHGE side chain), 1.05-0.74 (m, methyl of EHGE side chain).

Synthesis of NB-(PEHGE-NB)$_3$

Method D was used for the condensation reaction of HO-(PEHGE-OH)$_3$ ($M_{n,NMR}$ = 6,910 g mol^{-1}, 223 mg, 32.2 μmol) with *exo*-NB-COOH (35.8 mg, 259 mmol), DMAP (44.5 mg, 364 mmol), and EDC (76.5 mg, 399 mmol) in CH_2Cl_2 (2.2 mL) to give **NB-(PEHGE-NB)$_3$** as a pale yellow viscous liquid (172 mg). Yield: 71.4%

$M_{n,NMR}$ = 7,000 g mol^{-1}, $M_{n,SEC}$ = 7,690 g mol^{-1}, $Đ$ = 1.03

^{1}H NMR (400 MHz, $CDCl_3$): δ (ppm) 6.22-6.00 (m, -C*H*=C*H*- in norbornene ring), 5.23-5.01 (m, methine of PEHGE backbone at the chain end), 4.09 (s, -COOC*H*$_2$-), 3.67-3.58 (m, -$CH_2CH(C\mathit{H}_2$-)O-), 3.57-3.36 (m, -C*H*$_2$CH(CH_2-)O-, -CH_2C*H*(CH_2-)O-, -C(C*H*$_2$O-)$_3$), 3.34-3.21 (m, -$CH_2CH(CH_2OC\mathit{H}_2$-)O-), 3.10-2.98 (m, -C*H*CHCO- in norbornene ring), 2.90 (s, -C*H*CH_2CHCO- in norbornene ring), 2.32-2.11 (m, -C*H*CO- in norbornene ring), 2.02-1.84 (m, *exo*-H of -C*H*$_2$- in norbornene ring), 1.54-1.42 (m, methine of EHGE side chain), 1.42-1.17 (m, methylene of EHGE side chain), 0.99-0.62 (m, methyl of EHGE side chain).

Synthesis of trefoil-shaped PEHGE

Method E was used for the ROMO of NB-(PEHGE-NB)$_3$ ($M_{n,NMR}$ = 7,000 g mol^{-1}, 10.0 mg, 1.43 μmol, 170 μM in CH_2Cl_2) with G3 (7.6 mg, 8.6 μmol) in CH_2Cl_2 (143 mL) to give trefoil-shaped PEHGE as a colorless viscous liquid (7.3 mg). Yield: 71.9%

$M_{n,SEC}$ = 6,320 g mol^{-1}, $Đ$ = 1.05

^{1}H NMR (400 MHz, $CDCl_3$): δ(ppm) 6.54-1.82 (br, alkenyl of poly(norbornene) backbone), 3.68-3.58 (m, -$CH_2CH(CH_2$-)O-), 3.57-3.36 (m, -$CH_2CH(CH_2$-)O-, -$CH_2CH(CH_2$-)O-, -C(CH_2O-)$_3$), 3.35-3.19 (m, -$CH_2CH(CH_2OCH_2$-)O-), 1.55-1.43 (m, methine of EHGE side chain), 1.17-1.42 (m, methylene of EHGE side chain), 0.77-0.97 (m, methyl of EHGE side chain).

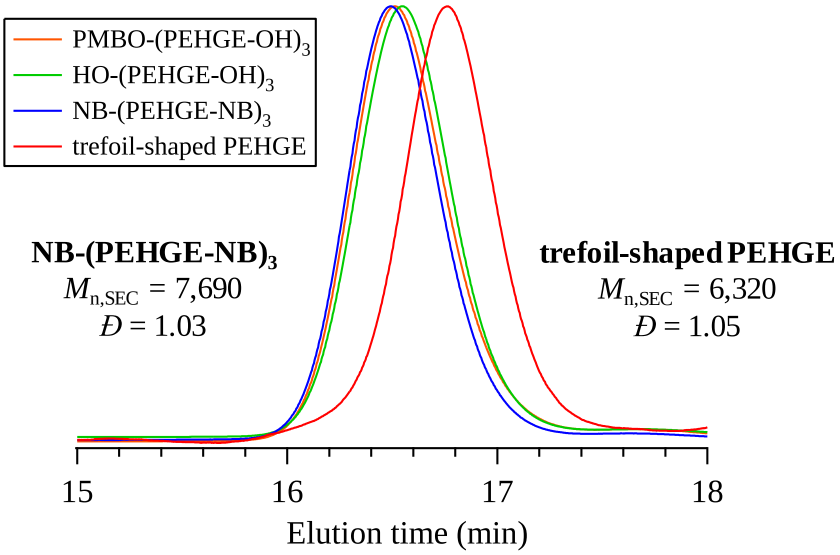

Supplementary Figure 19. SEC traces of PMBO-(PEHGE-OH)$_3$ (orange), HO-(PEHGE-OH)$_3$ (green), NB-(PEHGE-NB)$_3$ (blue) and trefoil-shaped PEHGE (red).

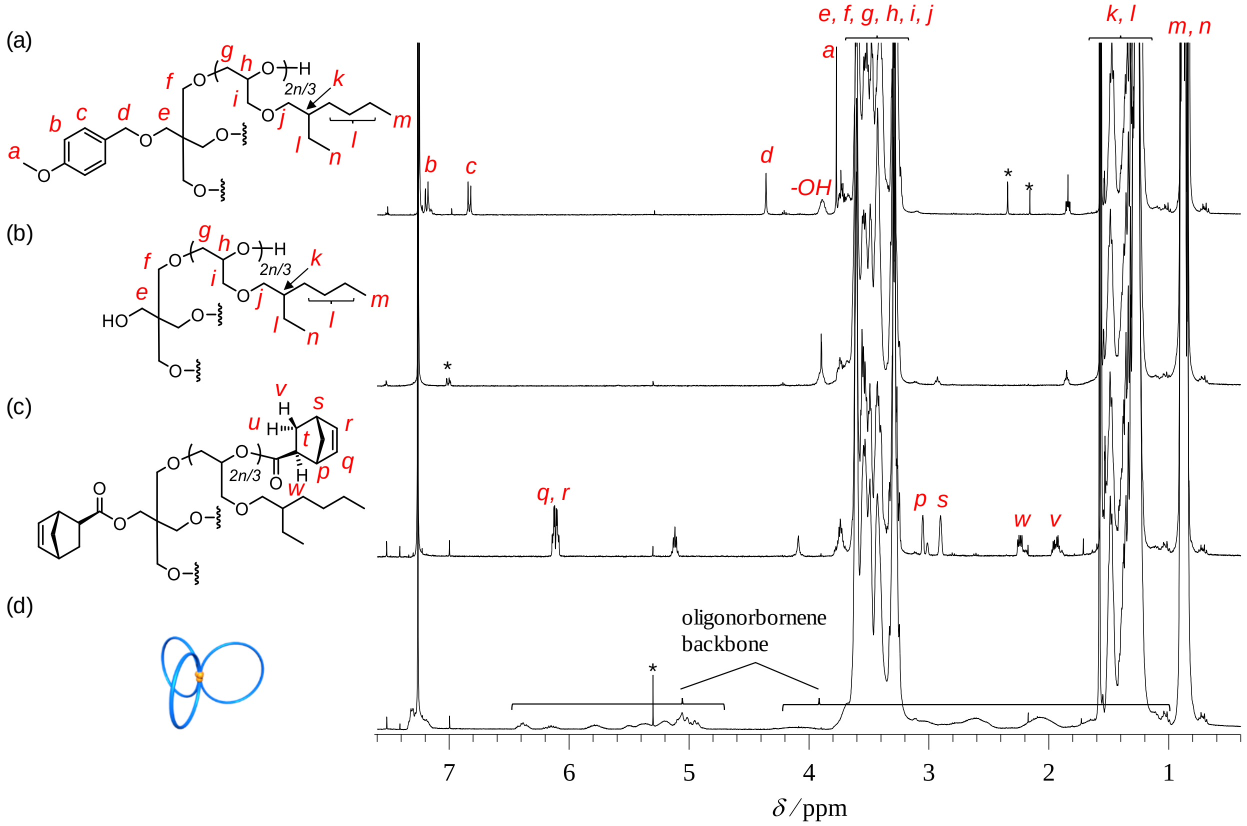

Supplementary Figure 20. ^{1}H NMR spectra of (a) PMBO-(PEHGE-OH)$_3$, (b) HO-(PEHGE-OH)$_3$, (c) NB-(PEHGE-NB)$_3$, and (d) trefoil-shaped PEHGE in $CDCl_3$ (400 MHz).

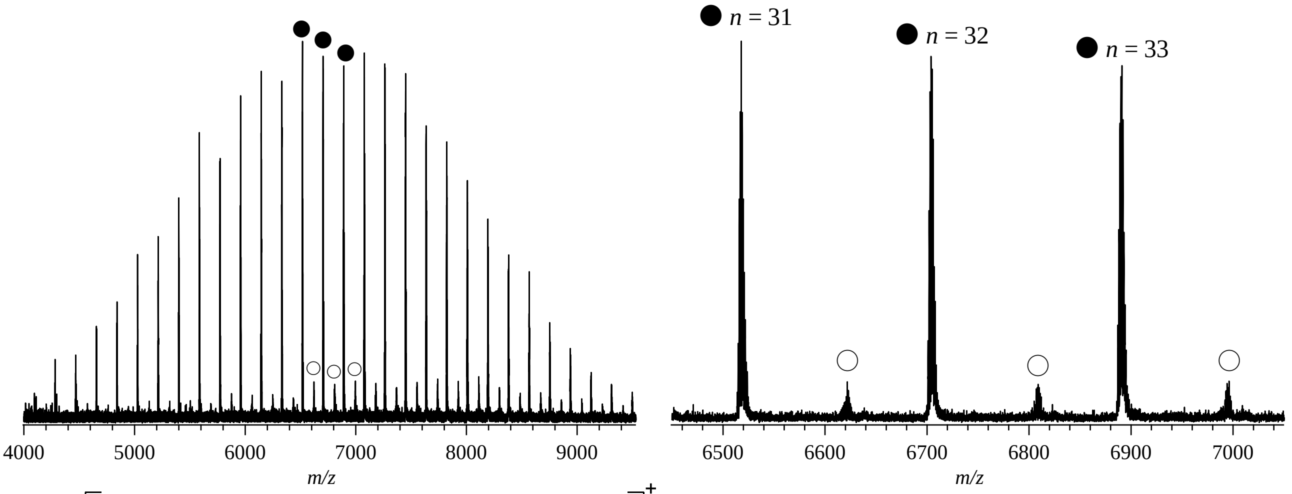

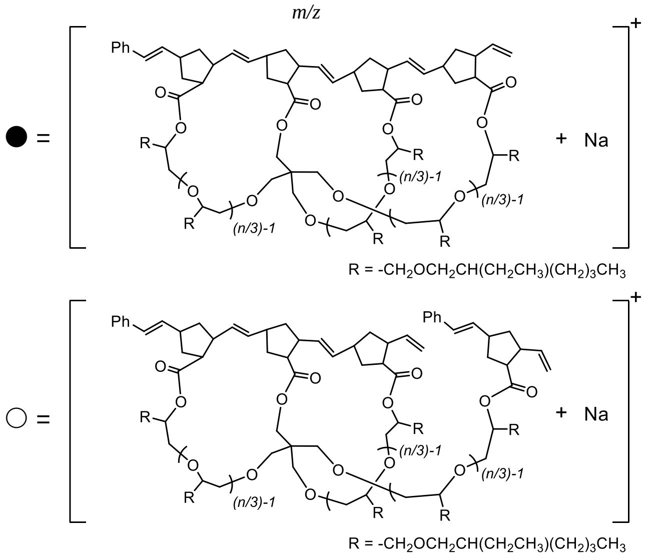

Calculated and observed M.W. of $[M + Na]^+$

	n = 31	n = 32	n = 33
Calcd.	6514.32	6700.48	6886.64
Obs.	6514.82	6700.95	6887.18

Supplementary Figure 21. MALDI-TOF MS analysis of trefoil-shaped PEHGE. The amount of twin-head tadpole product was approximately calculated to be 11.4% by the ratio of integrated peak heights.

Supplementary Table 4. Molecular characterization of trefoil-shaped PLLA, trefoil-shaped PEHGE and their precursors

Sample	$M_{n,NMR}$ [a] (g mol^{-1})	$M_{n,SEC}$ [b] (g mol^{-1})	$Đ$ [b]	yield (%)
PMBO-(PLLA-OH)$_3$	7,140	10,800	1.04	86.9
HO-(PLLA-OH)$_3$	7,150	10,900	1.04	44.3
NB-(PLLA-NB)$_3$	8,050	11,400	1.03	67.5
trefoil-shaped PLLA	-	7,700	1.09	91.4
PMBO-(PEHGE-OH)$_3$	6,560	7,490	1.03	63.0
HO-(PEHGE-OH)$_3$	6,910	7,390	1.03	55.6
NB-(PEHGE-NB)$_3$	7,000	7,690	1.03	71.4
trefoil-shaped PEHGE	-	6,320	1.05	71.9

[a] Determined by ^{1}H NMR. [b] Determined by SEC in THF using PSt standards.

S2. Additional results

S2-1. Systematic study on hydrodynamic volume, viscosity, and crystallinity

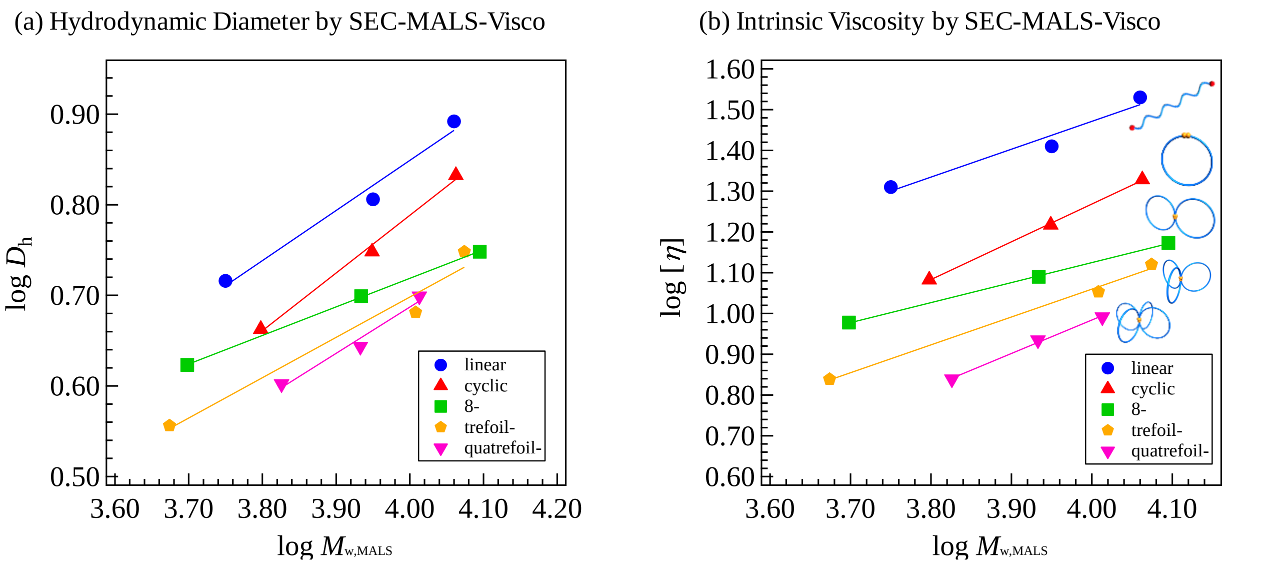

Supplementary Figure 22. Double logarithmic plots of $M_{\mathrm{w,MALS}}$ versus (a) D_{h} and (b) [η] for linear, monocyclic, 8-, trefoil-, and quatrefoil-shaped polymers (eluent, THF).

Polymer topology	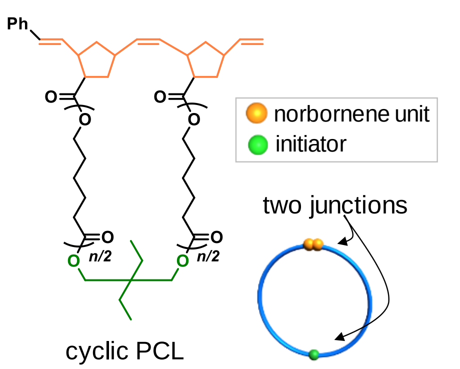	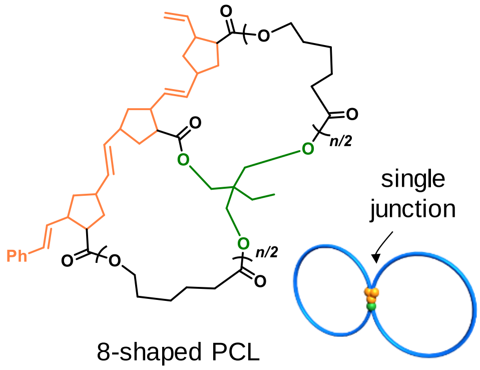
Possible crystalline formation	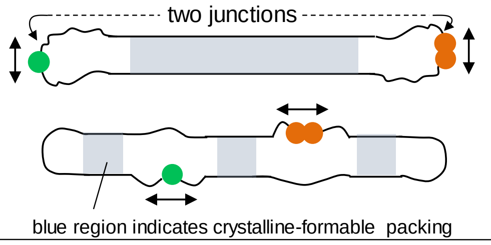	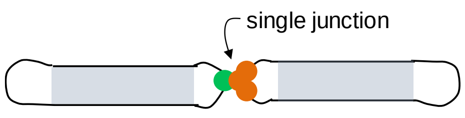
Chain packing disruption	Multiple	Diminished
T_{m} and crystallinity	Lower	Higher

Supplementary Figure 23. Illustration of proposed packing structure for the crystalline formation of cyclic PCL (left column) and **MC2**s (right column).

Supplementary Note 1. SAXS analysis

To further elucidate the topological effect on bulk properties of semicrystalline polymers, SAXS experiments was carried out for the *spiro*-multicyclic PCLs (**MC2-b**, **MC3-b**, and **MC4-b**) and their linear and cyclic counterparts (**linear$_{\mathbf{9k}}$** and **cyclic$_{\mathbf{9k}}$**) with molecular weight of ca. 9000. With respect to the SAXS analysis, the thicknesses of crystalline domain (d) were estimated by following equation:

$d = 2\pi/q^*$

where the q^* is primary scattering vector from the SAXS profiles as summarized in Supplementary Figure 24. Moreover, the crystalline lamellae thickness was determined by correlation function analysis according to the report using SasView.[4] The theoretical chain length of **MC2-b** per arm was calculated as 0.735 nm × ((($MW_{\mathbf{MC2\text{-}b}}$ – (MW_{NB} × 4 + MW_{ini})) ÷ 4), where 0.735 nm is the molecular length of a PCL repeating unit,[5] $MW_{\mathbf{MC2\text{-}b}}$ is $M_{n,NMR}$ of **MC2-b**, MW_{NB} is the molecular weight of oligonorbornene backbone, and MW_{ini} is the molecular weight of initiator.

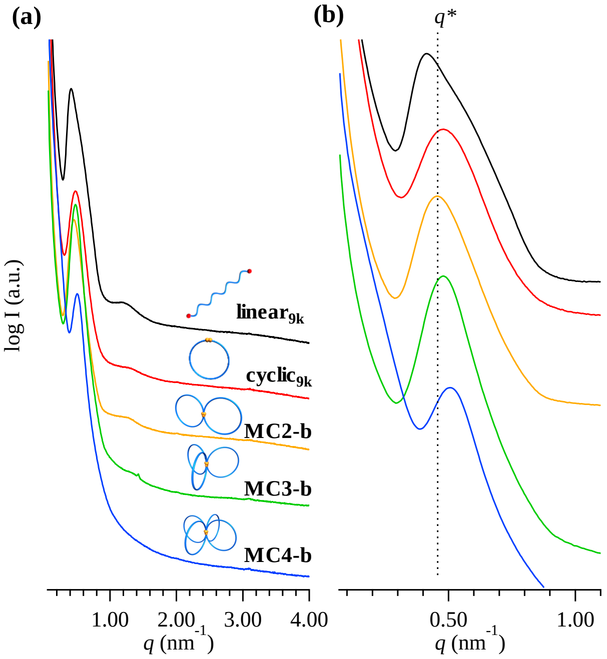

sample	SAXS peak analysis		Correlation function analysis	
	*q** (nm^{-1})	*d* (nm)	lamellae thickness (nm)	*d* (nm)
**linear$_{9k}$**	0.405	15.5	4.6	11.1
**cyclic$_{9k}$**	0.486	12.9	4.8	11.0
**MC2-b**	0.456	13.8	5.1	11.8
**MC3-b**	0.476	13.2	4.8	11.7
**MC4-b**	0.507	12.4	4.8	11.7

Supplementary Figure 24. (a) SAXS profiles of *spiro*-multicyclic PCLs (**MC2-b**, **MC3-b**, and **MC4-b**) and their counterparts (**linear$_{9k}$** and **cyclic$_{9k}$**) (bulk sample; SDD = 1.5 m). (b) expanded SAXS profiles for the determination of q^* and d, that shows obvious change of **MC2-b** compared to the topological counterparts.

S2-2. WAXD analysis

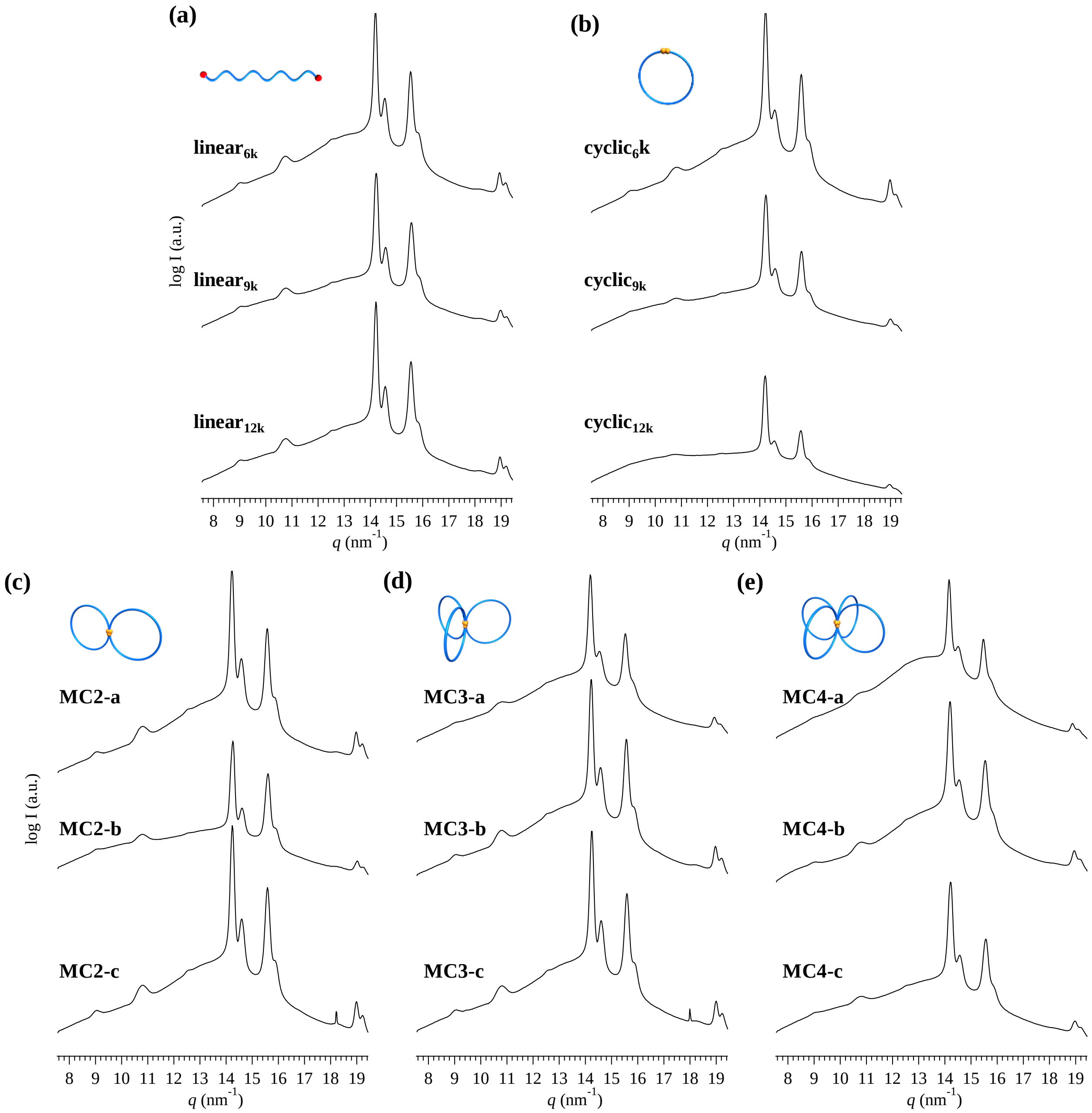

Supplementary Figure 25. WAXD profiles of (a) linear, (b) cyclic, (c) 8-shaped (**MC2**s), (d) trefoil-shaped (**MC3**s), and (e) quatrefoil-shaped PCLs (**MC4**s) with different molecular weight.

S2-3. DSC analysis

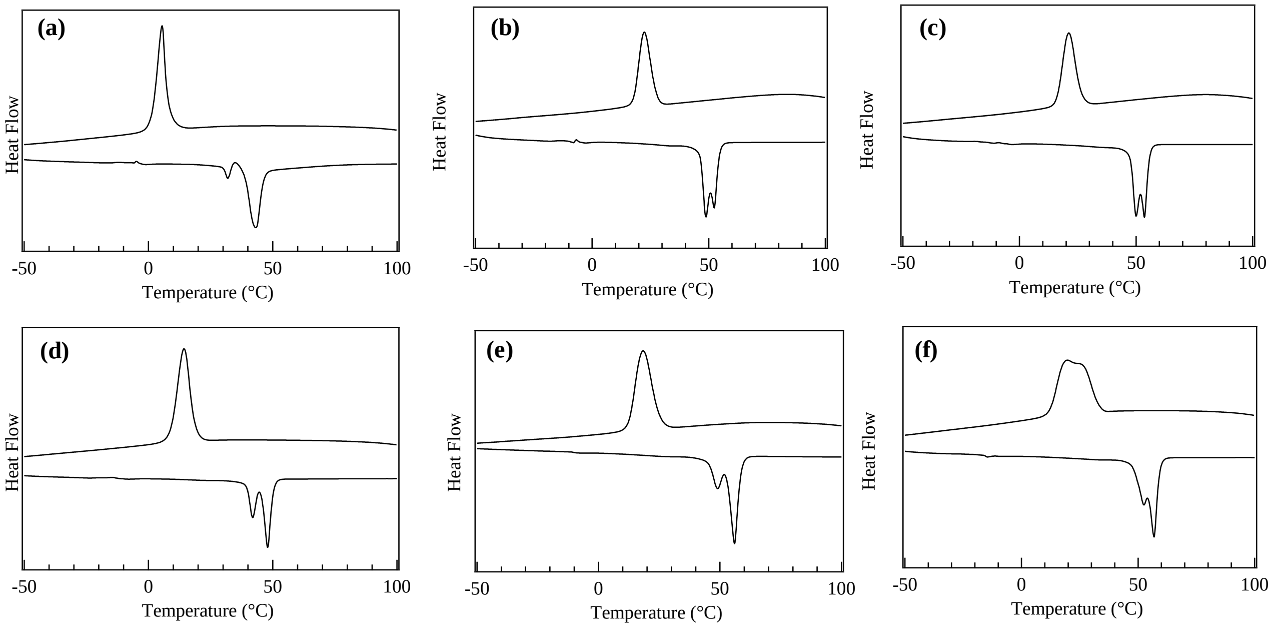

Supplementary Figure 26. DSC thermograms of (a) linear$_{6k}$, (b) linear$_{9k}$, (c) linear$_{12k}$, (d) cyclic$_{6k}$, (e) cyclic$_{9k}$, and (f) cyclic$_{12k}$.

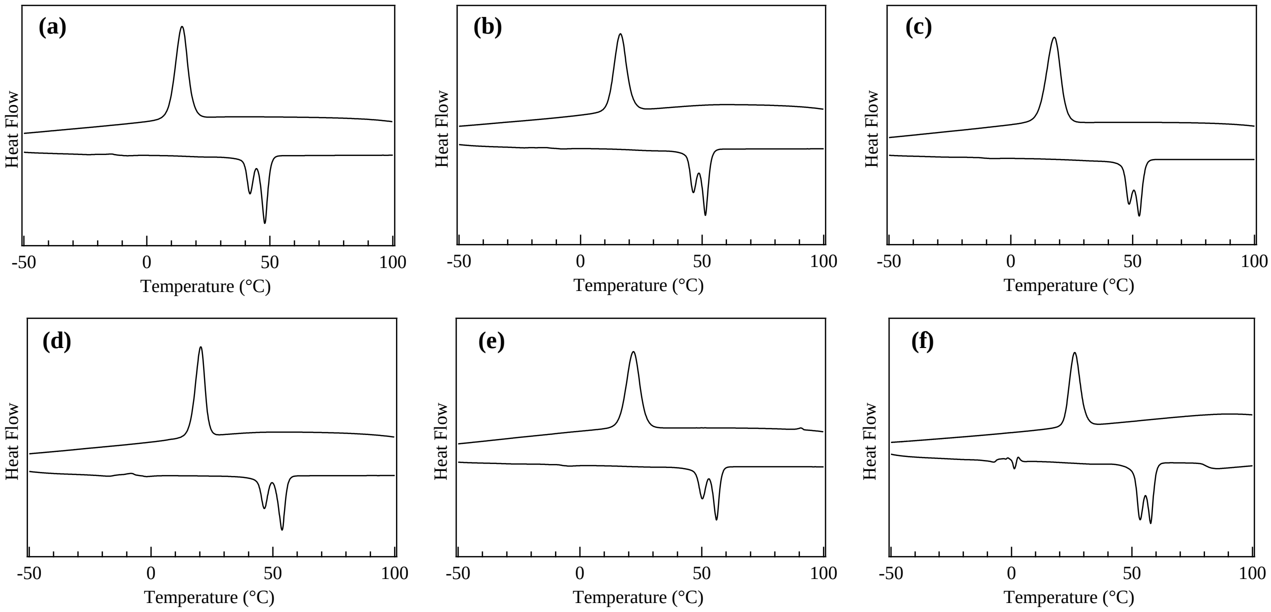

Supplementary Figure 27. DSC thermograms of (a) **P2-a**, (b) **P2-b**, (c) **P2-c**, (d) **MC2-a**, (e) **MC2-b**, and (f) **MC2-c**.

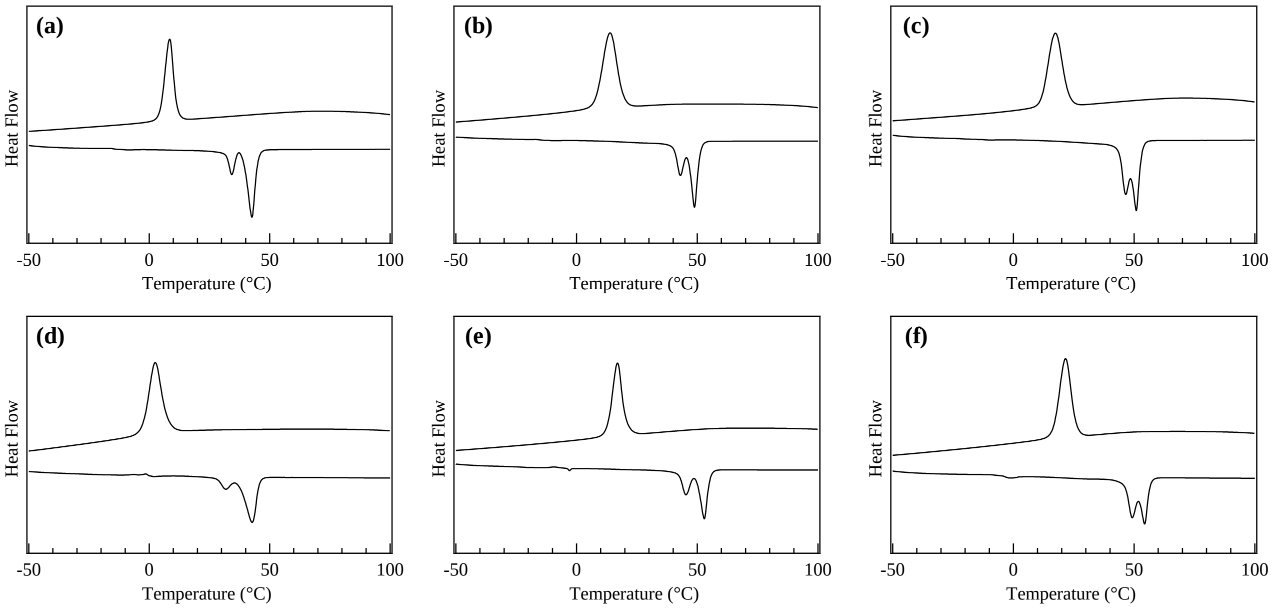

Supplementary Figure S28. DSC thermograms of (a) **P3-a**, (b) **P3-b**, (c) **P3-c**, (d) **MC3-a**, (e) **MC3-b**, and (f) **MC3-c**.

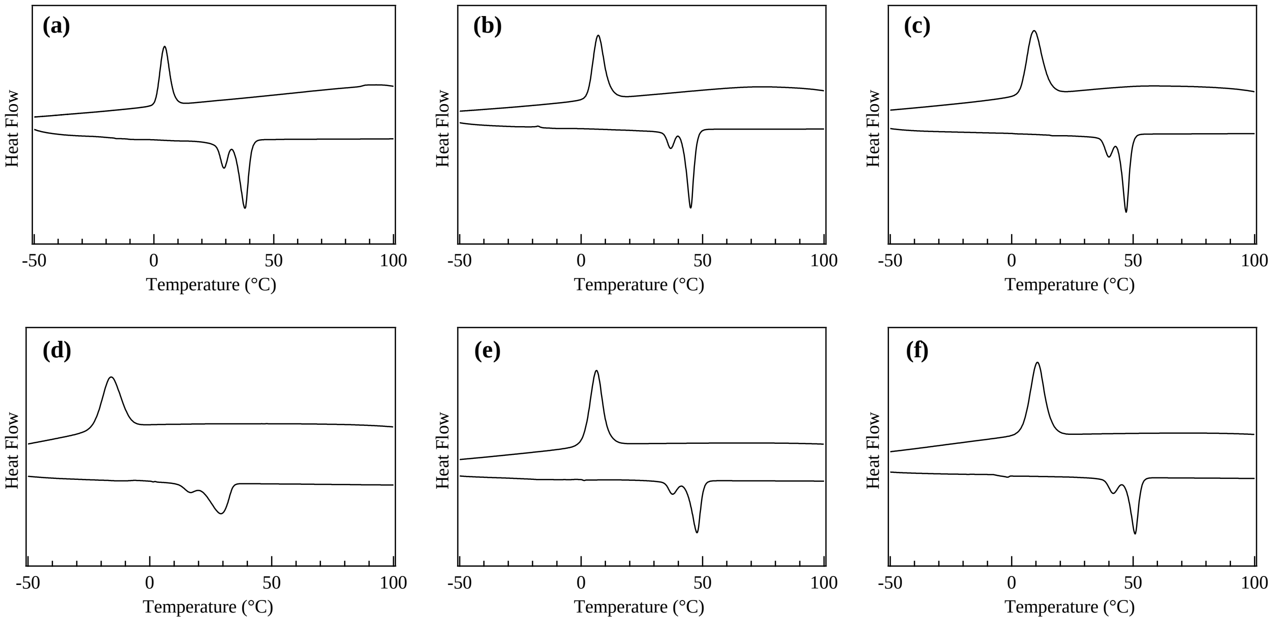

Supplementary Figure 29. DSC thermograms of (a) **P4-a**, (b) **P4-b**, (c) **P4-c**, (d) **MC4-a**, (e) **MC4-b**, and (f) **MC4-c**.

S2-4. TGA

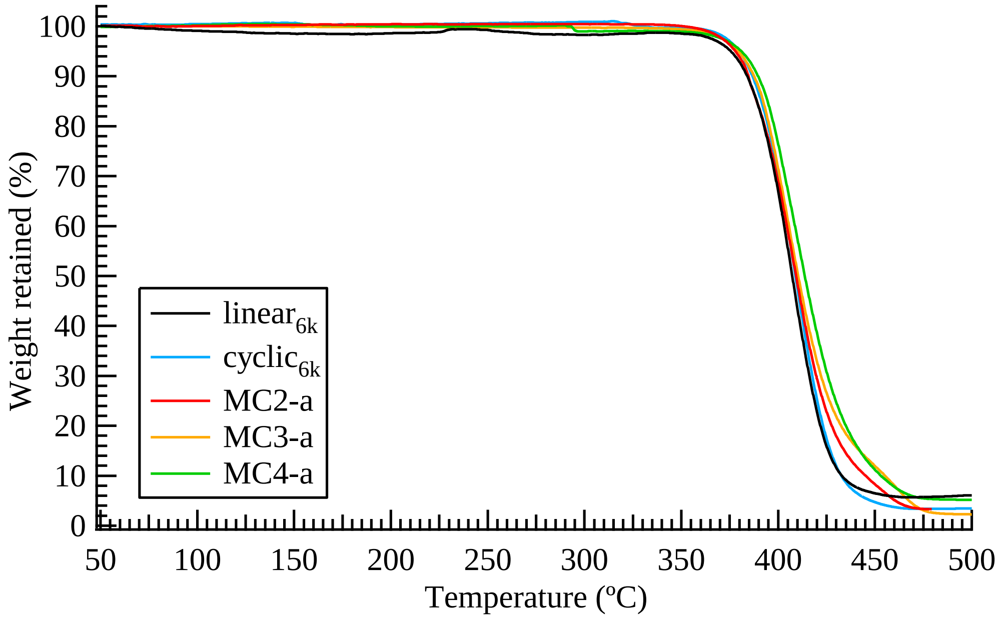

Supplementary Figure 30. TGA result of *spiro*-multicyclic PCLs and their linear and cyclic counterparts obtained under Ar atmosphere (total MW; ~6000 g mol^{-1}, heating rate; 10 °C min^{-1}).

Supplementary Table 5. Thermal degradation properties of *spiro*-multicyclic polymers and their linear and cyclic counterparts

Sample	$M_{n,NMR\ (precursor)}$ (g mol^{-1})	T_d (°C) [a]
$linear_{6k}$	6,540	384
$cyclic_{6k}$	6,540	387
MC2-a	6,200	389
MC3-a	6,160	388
MC4-a	6,620	390

[a] 10% degradation temperature (T_d) was determined by TGA.

S3. Supplementary References

1. Love, J. A.; Morgan, J. P.; Trnka, T. M.; Grubbs, R. H. A Practical and Highly Active Ruthenium-Based Catalyst that Effects the Cross Metathesis of Acrylonitrile. *Angew. Chem. Int. Ed.* **2002**, *41*, 4035–4037.
2. Satoh, Y.; Matsuno, H.; Yamamoto, T.; Tajima, K.; Isono, T.; Satoh, T. Synthesis of Well-Defined Three- and Four-armed Cage-shaped Polymers via "Topological Conversion" from Trefoil- and Quatrefoil-shaped Polymers. *Macromolecules* **2017**, *50*, 97–106.
3. Saha, B.; Choudhury, N.; Seal, S.; Ruidas, B.; De, P.; Aromatic Nitrogen Mustard-Based Autofluorescent Amphiphilic Brush Copolymer as pH-Responsive Drug Delivery Vehicle. *Biomacromolecules* **2019**, *20*, 546–557.
4. Strobl, G. R.; Schneider, M.; Direct Evaluation of the Electron Density Correlation Function of Partially Crystalline Polymers. *J. Polym. Sci. Part A-2, Polym. Phys.* **1980**, *18,* 1343–1359.
5. Isono T.; Miyachi K.; Satoh Y.; Nakamura R.; Zhang Y.; Otsuka I.; Tajima K.; Kakuchi T.; Borsali R.; Satoh T.; *Macromolecules* **2016**, *49*, 4178–4194.
